# Transcriptome analysis of the response to low temperature acclimation in *Calliptamus italicus* eggs

**DOI:** 10.1186/s12864-022-08705-3

**Published:** 2022-07-01

**Authors:** Qian Liu, Di Luo, Mengjia Wang, Xingmin Song, Xiaofang Ye, Roman Jashenko, Rong Ji

**Affiliations:** 1 International Center for the Collaborative Management of Cross-border Pest in Central Asia, Xinjiang Key Laboratory of Special Species Conservation and Regulatory Biology, Xinjiang Normal University, Urumqi, 830054 China; 2 Al-Farabi Kazakh National University, Almaty, Kazakhstan 050038

**Keywords:** *Calliptamus italicus*, Transcriptome, Low temperature acclimation, RNA interference, Cold tolerance

## Abstract

**Background:**

*Calliptamus italicus* is a dominant species in the desert and semi-desert grassland. It is widely distributed throughout many regions such as Asia, Europe, North Africa and the Mediterranean, and has enormous destructive potential for agriculture and animal husbandry. The *C. italicus* overwintering as eggs in the soil through diapause, and the cold tolerance of locust eggs is the key to their ability to survive the winter smoothly to maintain the population.

**Results:**

Transcriptome analysis of *C. italicus* eggs was carried out in this paper in constant low temperature acclimation, natural low temperature acclimation and room temperature. The differentially expressed genes related to cold tolerance were screened out, the differences in expression patterns under different low temperature acclimation were analyzed, and the genes in the significantly up-regulated pathways may play an important role in cold tolerance. The results show that different domestication modes can induce *C. italicus* eggs to express a large number of genes to alleviate low temperature damage, but *C. italicus* eggs are more sensitive to changes in temperature. Compared with the control, there are 8689 DEGs at constant low temperature and 14,994 DEGs at natural low temperature. KEGG analysis showed that DEGs were mainly enriched in pathways related to metabolism and biological systems under constant low temperature, and were mainly enriched in pathways related to biological systems and environmental information processing under natural low temperature. In addition, RNAi technology was used to further verify the regulation of genes in the significantly enriched up-regulated pathways on *C. italicus* eggs, and it was confirmed that the hatching rate of *C. italicus* eggs at low temperature was significantly reduced after interference.

**Conclusions:**

Transcriptome analysis of *C. italicus* eggs treated at different temperatures provided a theoretical basis for further understanding the adaptation mechanism of *C. italicus* eggs to low temperature. In addition, four potential RNAi target genes were verified in the eggs of *C. italicus* for the first time, providing new ideas for effective control of this species.

**Supplementary Information:**

The online version contains supplementary material available at 10.1186/s12864-022-08705-3.

## Introduction

Insects are poikilothermic who can survive in extreme low or high temperature that will affect their survival and individual development [1, 2]. The survival strategy of insects for adapting to the changes in external temperature has always been the core issue of insect evolution. Insects living in temperate and frigid regions are threatened by the low temperature in winter every year. In order to maintain the population and expand the distribution range, insects have formed a series of cold resistance mechanisms in the long-term evolution process [3]. For example, they adapt to the low-temperature by regulating related cold-resistance genes [4], synthesizing cold-resistant substances [5], increasing the expression of intracellular antifreeze proteins, and producing cold shock proteins [6]. On the other hand, many studies have reported that low-temperature acclimation can significantly improve the cold tolerance in insects [7, 8]. In general, these studies are carried out at a constant low temperature because they are simpler to operate and only require standardized comparison methods [9]. However, the natural environment is not stable. It has diurnal and seasonal changes in temperature and photoperiod. Therefore, the stress response under constant low temperature acclimation cannot fully explain the cold tolerance mechanism of insects in their natural environment against low temperature [10]. In nature, long-term seasonal temperature fluctuations will have a natural domestication effect on insects, which is an adaptive response with regard to the decrease in the seasonal temperature [11]. Combining the cold tolerance molecular mechanisms of insects under constant and natural low temperature domestication can reflect the characteristics of insect to cold tolerance, and provide a theoretical basis for revealing the insect’s life history countermeasures, physiological and biochemical modulation, and selective evolution mechanisms.

Transcriptome technology can identify the main control genes and secondary change genes under specific conditions. Transcriptome sequencing of insects under different conditions, such as low temperature, dehydration, starvation and pathogenic fungi infection, will help in comprehensively revealing the molecular mechanism of related gene functions and phylogenetic evolution in their life activities [12–14]. Li et al. [15] analyzed the transcriptome of *Ceracris kiangsu* and found that the body significantly up-regulated genes related to stress response and ATP production in response to low temperature stress. Dunning et al. [16] studied the transcriptome of micrarchus alpine and lowland population and found that temperature is an important factor driving the evolution of micrarchus species. In addition, transcriptome is also commonly used to identify RNA interference targets and provide strategies for functional verification and pest control [17–19].

*Calliptamus italicus* belongs to Orthoptera, Catantopidae, Callipamus and it is widely distributed in Central Europe, North Africa, Central Asia, the western part of the Siberian Plain, the northwestern part of Mongolia, the eastern and northern parts along the Mediterranean Sea, etc. In China, *Calliptamus italicus* is mainly distributed in desert and semi-desert grasslands in the north of Xinjiang at an altitude of 800–2300 m [20, 21], which can harm up to 17 families and 45 species of plants, and cause serious harm to the development of animal husbandry and agriculture in Xinjiang [22]. The *C. italicus* overwintering as eggs in the soil through diapause, and the development of overwintering eggs undergoes three stages: Early-development, Diapause, and Diapause-terminated [23]. Xinjiang is cold in winter with an average temperature of − 14.5 °C, the extreme temperature reaches around − 40 °C [24]. The cold tolerance of locust eggs is the key to their ability to survive the winter smoothly to maintain the population. The cold tolerance of insects is a biological process controlled by multiple factors. A generally accepted view is that low-temperature acclimation can improve cold tolerance but we have limited understanding of the regulation mechanism of cold tolerance. Previous studies have found differently responded mechanisms for different organisms under constant low-temperature acclimation. Transcriptome analysis of *Blattella germanica* [25] and *Microdera punctipennis* [26] after low-temperature acclimation at 4 °C, it was found that low-temperature response genes in *Blattella germanica* were functionally enriched in carboxylic acid metabolism, stress response, and carbohydrate metabolism, whereas genes in *Microdera punctipennis* was mainly involved in metabolic pathways, such as purine metabolism, thiamine metabolism and glycolysis/gluconeogenesis. Also, there is the different response of organisms to various modes of low-temperature acclimation. Analysis of transcriptome of *Ericerus pela* with a natural acclimation condition suggested that the majority of genes were enriched in the process of signal transduction and metabolism, and the expression of antifreeze related genes, such as heat shock protein (HSP) and anti-freeze protein (AFP), were up-regulated [27]. Under a slow-cooling mode of condition, the differentially expressed genes (DEGs) of *Trifolium ambiguum* were significantly enriched in photosynthesis, photosynthesis-antenna proteins, and starch and sucrose metabolism, whereas when treated with a sudden-cooling mode, the DEGs were significantly enriched in starch and sucrose metabolism, sesquiterpenoid and triterpenoid biosynthesis, and flavonoid biosynthesis [28].

These studies show that DEGs are mainly enriched in pathways related to metabolism, transcription and environmental signal processes after low temperature stress. Genes directly related to low temperature stress, such as hsp, afp and enzyme genes, are differentially expressed. However, the genes or pathways related to cold tolerance have not been validated, and the previous studies mentioned only used one temperature as a stress or adaptation temperature in their study. However, insects have different responses to different low temperatures. Therefore, this experiment constructed transcriptome sequencing under constant and natural low temperature acclimation, identified candidate genes related to cold tolerance through differential expression analysis, and verified RNAi target genes related to cold tolerance through dsRNA artificial injection test, in order to explore the molecular biological mechanism of *C. italicus* eggs resisting low temperature in winter.

## Results

### Transcriptome sequencing and functional annotation

The *C. italicus* eggs at the early-development, diapause and diapause-terminated stages were sequenced at constant low temperature acclimation (0 °C), natural low temperature acclimation and room temperature (27 °C) conditions, respectively. Three replicates were conducted for each treatment and each stage, resulting in construction of 27 cDNA libraries (Table 1). Transcriptome sequencing yielded 6.40G–7.33G of clean reads for single sample with a GC content of 43.24–47.05%, which showed a small deviation with random distribution. The percentage of bases >Q30 was greater than 92.35%, indicating that the quality of this sequencing data was reliable and could be used for further analysis.

**Table 1:** Data quantity statistics of *C. italicus* eggs samples before and after filtration

Sample	Raw Reads	Clean Reads	Total mapped	GC Content(%)	Q30(%)
T_ED	23,427,576	6.82G	37,991,977(83.58%)	44.52	92.98
T_D	24,603,027	7.24G	40,259,910(83.36%)	43.50	94.81
T_DT	23,000,542	6.53G	35,164,138(80.81%)	44.04	94.64
Z_ED	22,897,560	6.66G	37,441,784(84.41%)	47.05	92.71
Z_D	23,772,541	6.95G	37,341,301(80.50%)	43.59	94.44
Z_DT	22,450,927	6.40G	35,076,205(82.11%)	43.24	94.72
N_ED	24,035,594	6.99G	38,930,646(83.59%)	46.47	92.87
N_D	25,000,086	7.33G	40,111,145(82.05%)	43.43	94.47
N_DT	22,919,680	6.56G	35,997,595(82.36%)	45.02	93.83

Note: T (Twenty-seven) represents the treatment in the artificial climate chamber at 27 °C; Z (Zero) represents the treatment at 0 °C low temperature acclimation for 15 days; N (Natural) represents the treatment under natural outdoor conditions. ED (Early-development) stands for early developmental stage; D (Diapause) stands for diapause stage; DT (Diapause-terminated) stands for diapause release stage. Raw reads: raw sequence data; Clean reads: the number of sequencing sequences multiplied by the length of the sequence, and converted to G as the unit; Total mapped: statistics of sequencing sequences that can be located on the genome; GC content: bases G and The number of C accounts for the percentage of total bases; Q30: the percentage of bases with a Phred value greater than 30 to the total bases

Transcriptome data of *C. italicus* was annotated by using seven databases including Nr, Nt, KEGG, Swiss-Prot, PFAM, GO, and KOG((Table 2). The large number of unigenes (78,623) could be annotated by NR, accounting for 32.23% of the total unigenes. KEGG provided annotation for 16.60% of the total unigenes, followed by GO and PFAM (30% of unigenes). A least number of unigenes (16,777) were annotated by KOG, accounting for 6.87% of the total unigenes. A total of 9149 unigenes could be annotated in all seven databases, accounting for 3.86% of the total unigenes.

**Table 2:** Statistics of the functional annotations of the unigenes of *C. italicus* eggs

Annotated databases	Number of unigene hits	Percentage
Nr	78,623	32.23%
Nt	31,633	12.97%
KEGG	40,485	16.60%
Swiss-Prot	35,894	14.71%
PFAM	56,106	23.00%
GO	56,096	23.00%
KOG	16,777	6.87%
Annotated in all Databases	9419	3.86%
All	243,877	100%

### Statistical analysis of differentially expressed genes (DEGs)

**Fig. 1:**
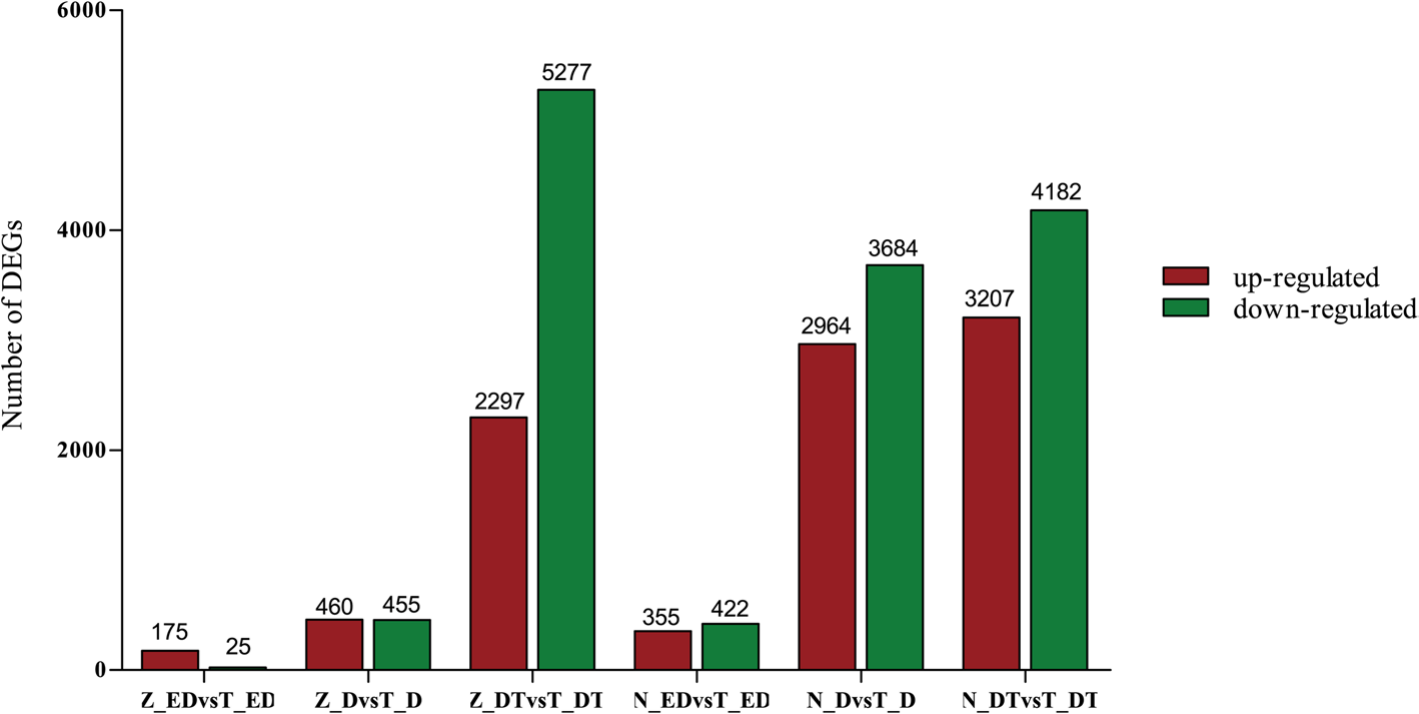
Analysis of DEGs in different acclimation groups of *C. italicus* egg. Up-regulated DEGs (red), and down-regulated DEGs (green) were presented by histogram

The statistics of the number of DEGs under different treatments and stages are shown in Fig. 1(Table S1). Samples in the constant low-temperature(Z vs T) and the natural low-temperature acclimation(N vs T) group showed 200 and 777 DEGs in the early-development stage, respectively. They had 915 and 6828 DEGs in the diapause stage, and 7574 and 7389 DEGs in the diapause-terminated stage. The number of DEGs increased with the development of *C. italicus* eggs. The number of up-regulated genes at early-development and diapause stage in the constant low-temperature acclimation group was more than that of the down-regulated genes, while an opposite phenomenon was found for DEGs at diapause-terminated stage. For natural low-temperature acclimation group, the number of down-regulated genes was more than that of the up-regulated genes.

**Fig. 2:**
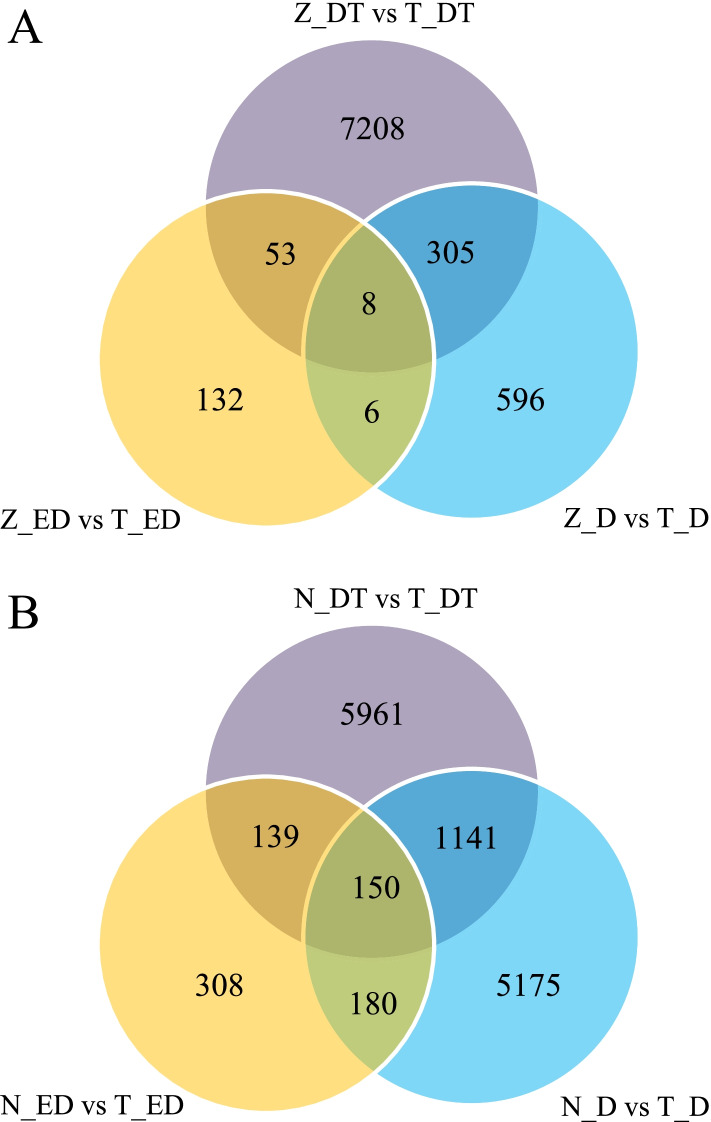
DEGs Four-way Venn diagram in different acclimation groups of *C. italicus* egg

A venn diagram was created based on further comparison of the DEGs between constant low-temperature and natural low-temperature acclimation group. The results showed that eight co-expressed genes were found in the constant low-temperature acclimation group at all stages, and 132, 596 and 7208 specifically expressed genes were detected at the early-development, diapauses, and diapause-terminated stage, respectively (Fig. 2,A). In the natural low-temperature acclimation group, 150 co-expressed genes were found at all stages, and 308, 5175 and 5961 specifically expressed genes were detected in the early-development, diapauses, and diapause-terminated stage, respectively (Fig. 2,B).

### GO and KEGG annotation of DEGs

GO enrichment analysis of all DEGs in the constant low-temperature acclimation group showed that 31 significantly enriched terms were annotated (Fig. S1,A), among which the largest number of DEGs (803) fell into “protein metabolic process” in the biological process. Whereas 1093 and 2389 genes were annotated by “organelle” in cellular component and “binding” in molecular function, respectively (Table S2). GO enrichment analysis of all DEGs in the natural low-temperature acclimation group showed that 13 significantly enriched terms were annotated (Fig. S1,B), among which the largest number of DEGs (145) were annotated as “cellular response to stress” in biological process, while 361 DEGs were annotated by “transition metal binding” in molecular function, and no significantly enriched DEGs were found in the cellular component (Table S3).

KEGG analysis showed that a large number of DEGs in the constant low-temperature acclimation group were enriched in 64, 167 and 290 pathways, of which 13, 13 and 18 pathways had significantly enriched DEGs at the three stages, respectively [29–31] (Fig. S2,A-C). They were mainly related to metabolism, environmental adaptation and signal transmission, such as arginine and proline metabolism, Circadian rhythms, and FoxO signaling pathway (Table S4). In the natural low-temperature acclimation group, a large number of DEGs were enriched in 147, 285 and 289 pathways respectively, of which 18, 20 and 27 pathways were significantly enriched with DEGs (Fig. S2,D-F), such as insulin signaling pathway, AMPK signaling pathway, and protein digestion and absorption (Table S5).

### Validation of DEGs by RT-qPCR

**Fig. 3:**
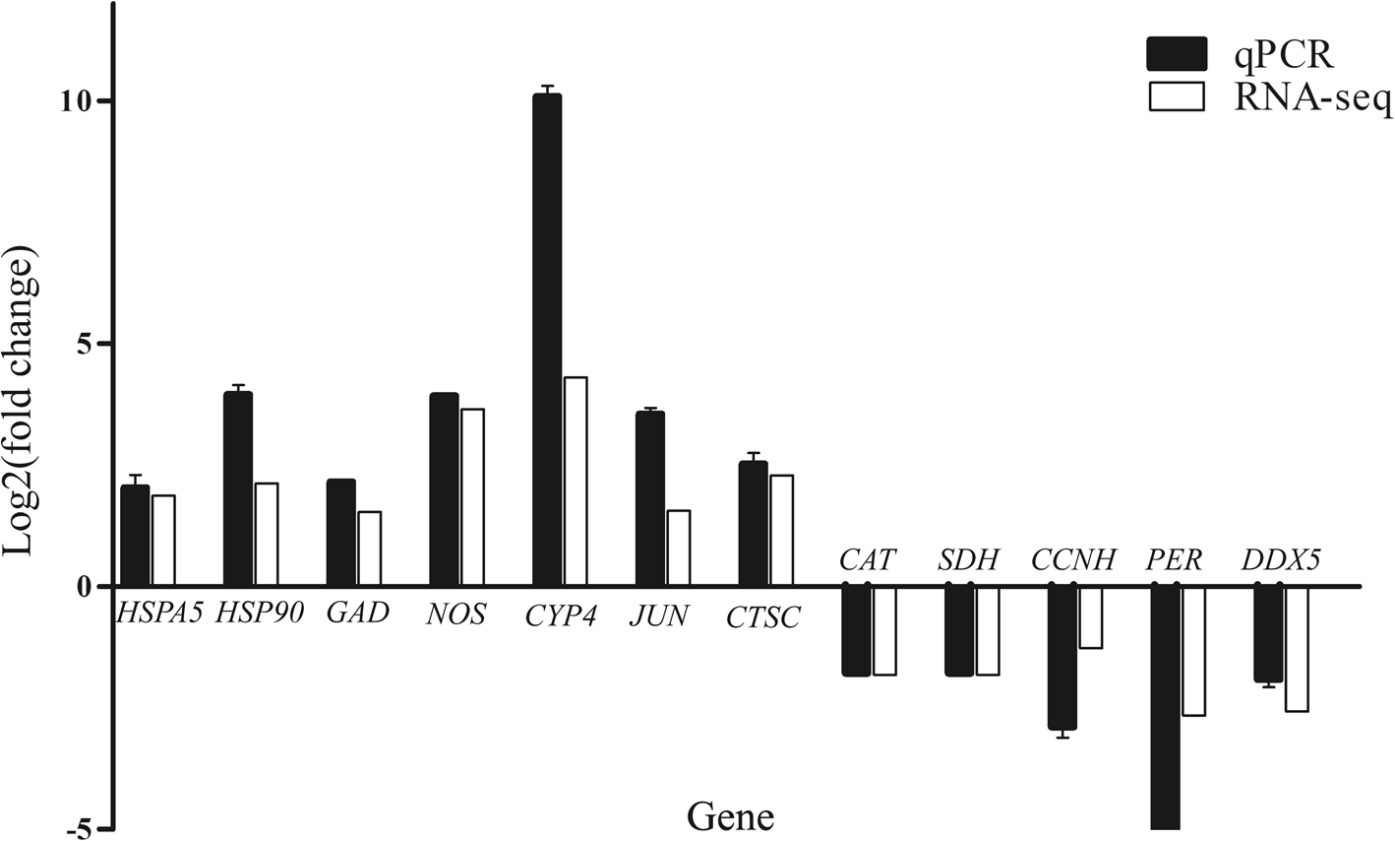
RNA-seq data validation by quantitative real-time PCR (RT-qPCR). The histograms show 12 DEGs of *C. italicus* egg

In order to evaluate the validity of transcriptome data, the expression of DEGs screened above was analyzed by RT-qPCR (Fig. 3). The results showed that the expression pattern of DEGs analyzed by qPCR was basically consistent with that reflected by RNA-seq, thus indicating the reliability of the RNA-seq results (Table S6).

### Identification of cold-tolerance related genes and RNA interference (RNAi) verification

Further analysis of significantly enriched 10 up- and down-regulated pathways under two low-temperature acclimation treatments suggested that the up-regulated pathways were significantly enriched under constant low-temperature which is mainly related to metabolism (Table 3). Among them, the largest numbers of genes were involved in amino acid metabolism, such as *GAD*, *NOS*, *OAT*, *ALDH*, and *TH*. Two significantly down-regulated pathways, the longevity regulation pathway and MAPK signaling pathway, were enriched with the largest numbers of genes, such as *SOD*, *HSPA1_8*, *CRYAB*, *CAT*, and *JUN*. The up-regulated pathways significantly enriched under natural low-temperature acclimation were mainly related to organismal systems (Table 4). Among them, pathways like that of the insulin signaling pathway and Circadian rhythms processed the largest number of enriched genes: *FASN*, *RKAR*, *CALM*, and *PKA*. Among the significantly down-regulated pathways, citrate cycle (TCA cycle) and AMPK signaling pathway were enriched with the most abundant genes: *SDH*, *PCK*, and *FOXO3*.

**Table 3:** 10 significantly enriched up- and down-regulated pathways in the constant low temperature acclimation group (Z vs T)

	Term	ID	Input number	Up/ down	*P*-Value	KO_name
Genetic Information Processing	Ribosome	ko03010	22	up	4.00E-14	RPL27|RPS5|RPL36|RPS24|RPS28|RPS6|RPL35A|RPL18|RPL13|RPL32|RPL6|RPS27A|RPS3|RPS25|RPL7|RPS23|RPL12|RPS16|RPS12|RPL8|RPS21|RPLP2|
Metabolism	Taurine and hypotaurine metabolism	ko00430	2	up	0.001598	GAD|GAD|
Metabolism	Arginine and proline metabolism	ko00330	3	up	0.008234	NOS|rocD,OAT|ALDH|
Metabolism	Tyrosine metabolism	ko00350	6	up	0.011154	DDC|TH|FAH|adhP|HPD|adhP|
Metabolism	beta-Alanine metabolism	ko00410	7	up	0.015515	GAD|CNDP2|ALDH|ABAT|ALDH6A1|ABAT|ALDH|
Organismal Systems	Thyroid hormone synthesis	ko04918	2	up	0.034976	HSPA5,BIP|gpx|
Organismal Systems	Circadian rhythm	ko04710	4	down	0.000319	PRKAB|CSNK1E|ARNTL,BMAL1,CYC|PER|
Organismal Systems	Longevity regulating pathway - multiple species	ko04213	32	down	0.001792	SOD1|HSPA1_8|ADCY2|CRYAB|CAT|CRYAB|CRYAB|CRYAB|SOD1|SOD1|CRYAB|ADCY8|HSPA1_8|INSR,CD220|AKT|HSPA1_8|ADCY2|CRYAB|FOXO3|SOD1|CRYAB|SIRT1|HSPA1_8|CRYAB|HSPA1_8|CRYAB|HSPA1_8|HSPA1_8|E4.6.1.1|EIF4EBP2|AMPK|HSPA1_8|
Environmental Information Processing	MAPK signaling pathway	ko04010	34	down	0.04892	ATF4|HSPA1_8|CACNB2|JUN|ALK5|RPS6KA|RSK2|LAMTOR3|CDC42|PTPRR|MNK|DUSP10|HSPA1_8|AKT|RASGRP3|MAX|HSPA1_8|MAP 2 K5|DUSP|MAX|NFKB1|MAP 3 K2|HSPA1_8|CRK|CREB2|ATF4|PLA2G4,CPLA2|HSPA1_8|FLNA|RAC1|HSPA1_8|HSPA1_8|CREB2|HSPA1_8|
Metabolism	Oxidative phosphorylation	ko00190	1	down	0.049903	ATP4|

**Table 4:** 10 significantly enriched up- and down-regulated pathways in the natural low temperature acclimation group(N vs T)

	Term	ID	Input number	Up/ down	P-Value	KO_name
Cellular Processes	Signaling pathways regulating pluripotency of stem cells	ko04550	8	up	5.15E-08	ATBF1|FZD9_10|ATBF1|FZD4|ATBF1| ISL1|ATBF1|WNT1|
Organismal Systems	Protein digestion and absorption	ko04974	18	up	5.25E-05	PRSS1_2_3|NCX|PEPT1|COL9A|COL18A|COL5AS|DPP4|PEPT1|COL18A|NHE3|COL4A|COL2A|COL13A|COL4A|CD26|SLC16A10|COL5AS|KCNQ1|
Organismal Systems	Circadian entrainment	ko04713	13	up	0.001528	NOS1|GRIN1|PLCB|ADCY9|CALM|PKA|PLCB|GNG13|PRKCA|ADCY5|PRKG1|ADCY1|CALM|
Organismal Systems	GABAergic synapse	ko04727	7	up	0.002846	GAD|GNAI|ADCY9|PRIP|GAD|NSF|VGAT|
Organismal Systems	Insulin signaling pathway	ko04910	20	up	0.006257	JNK|FASN|FASN|FASN|PRKAR|FASN|PPP1C|CALM|PKA|INS|FASN|BRAF|FASN|FASN|FASN|FASN|CALM|PIK3CA_B_D|FASN|FASN|
Environmental Information Processing	PI3K-Akt signaling pathway	ko04151	5	up	0.021109	HSP90|COL4A|BRCA1|COL4A|LAMC1|
Environmental Information Processing	AMPK signaling pathway	ko04152	31	down	0.001212	EEF2|PFKFB4|CAMKK2|PFKFB2|CREB3|SCD|EEF2|EEF2|PCK|PFKFB1|PFKFB2|EEF2|IRS1|PDPK1|PPP2R1|PPP2C|SREBF1|CD220|AKT|EEF2|EEF2|EEF2|CCNA|FBP|FOXO3|RHEB|SCD|SIRT1|PFK|SCD|desC|
Organismal Systems	Longevity regulating pathway - multiple species	ko04213	24	down	0.001681	HSPA1_8|CRYAB|CRYAB|CRYAB|CRYAB|SOD1|HSPA1_8|IRS1|E4.6.1.1|HSPA1_8|CD220|FOXA2|AKT|HSPA1_8|FOXO3|SIRT1|CRYAB|HSPA1_8|CRYAB|HSPA1_8|HSPA1_8|HSPA1_8|EIF4EBP2|HSPA1_8|
Metabolism	Glutathione metabolism	ko00480	15	down	0.012074	ANPEP|HPGDS|GGCT|SMS|HPGDS|CD224|GST|HPGDS|GST|HPGDS|GST|HPGDS|GST|CD13|ANPEP|
Metabolism	Citrate cycle (TCA cycle)	ko00020	26	down	0.018255	SDHA|OGDH|LSC2|PC|CS|ACO|MDH2|aceF|IDH1|IDH2|IDH3|ACLY|PCK|SDH1|SDH2|SDH3|SDH4|icd|fumC|sucA|lpd|pdhD|SDHD|SDHB|DLD|DLAT|

Many studies on insects have shown that the DEGs in response to temperature stress were mainly enriched in the pathways related to low-temperature regulation. These pathways can be summarized under three aspects. Firstly, cold-regulation signal transduction [32, 33], such as MAPK signaling pathway, PI3K-Akt signaling pathway, and Calcium signaling pathway; Secondly, cold-resistant physiological metabolism [34], such as arginine and proline metabolism, Cytochrome P450 metabolism, and oxidative phosphorylation; Thirdly, the environmental adaptation [4], such as Circadian rhythms. Along with this study results and previous reports related to gene regulation at low-temperature stress [35–37], four genes related to low temperature tolerance (*Hsp90*, *HSPA5*, *NOS* and *GAD*) were screened from the significantly up-regulated pathways of the two comparison groups, and the role of these four genes in the low-temperature tolerance of *C. italicus* eggs was verified by RNAi technology.

### dsRNA treatment of cold resistant genes

**Fig. 4:**
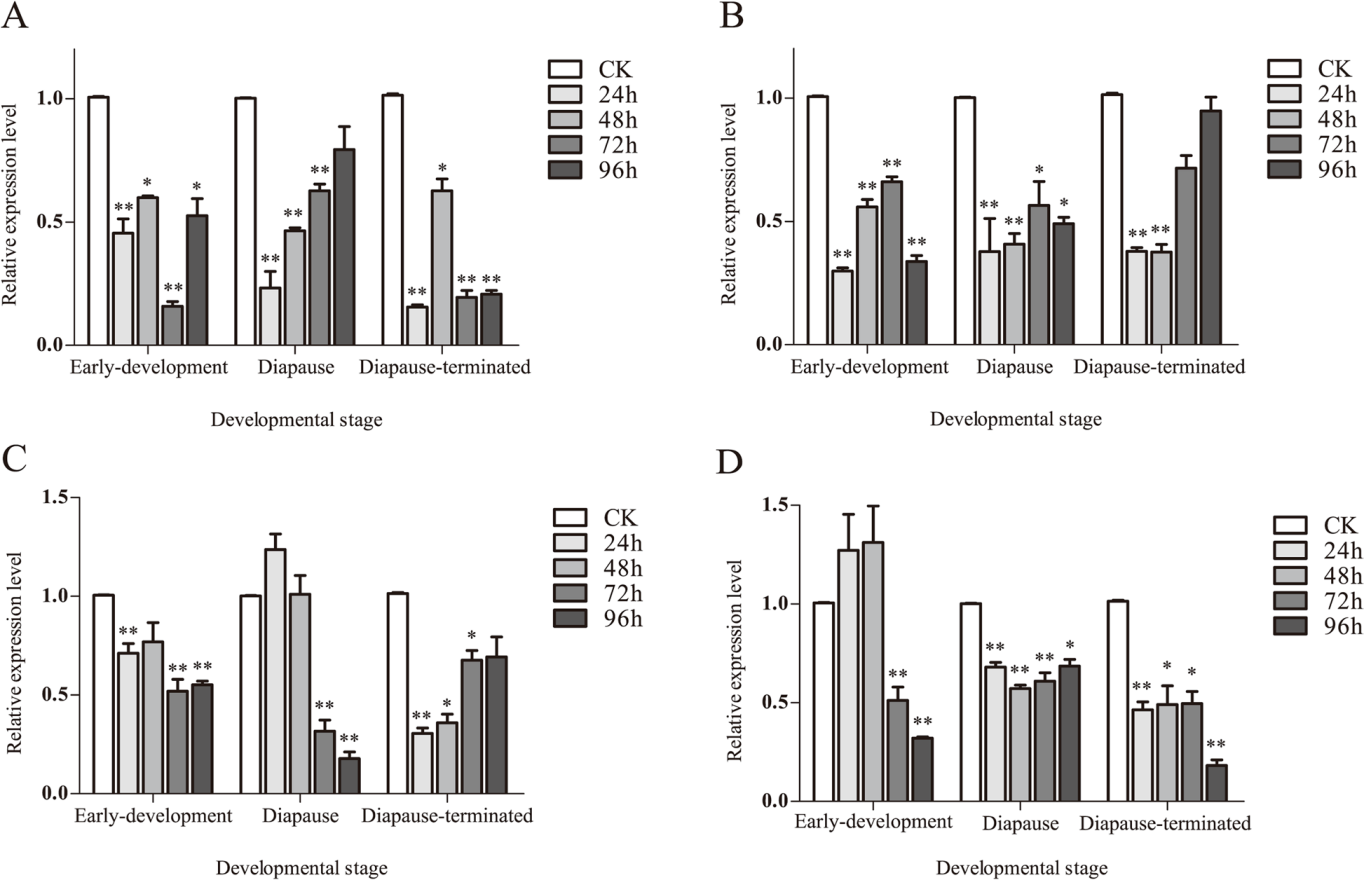
RT-qPCR analysis of *Hsp70*,*Hsp90*,*GAD* and *NOS* from *C. italicus* egg after RNAi at different times

Compared with the control, significantly different expression was found after injection of dsRNA, but the interference efficiency was different among the treatment groups at different times (Fig. 4, Table S7). A highest interference efficiency of 84.2% was recorded for early-development stage after injection of dsHsp70 for 72 h, whereas the lowest interference efficiency of 40.15% was detected after injection for 48 h. The injection for 24 h showed the highest interference efficiency of 76.7% at diapause stage, which gradually decreased, and the lowest interference efficiency was only 20.7% at 96 h after injection. At diapause-terminated stage, the interference efficiency was the highest (84.5%) at 24 h after injection, and was the lowest (37.4%) at 48 h upon injection. After injection of dsHsp90, the interference efficiency of early-development stage and diapause stage reached the greatest value of 70.1 and 66.2%, respectively at 24 h, and then decreased gradually, with the lowest interference efficiency of 34 and 43.6% at 72 h, respectively. The highest interference efficiency (62.4%) was detected at the diapauses-terminated stage at 48 h of treatment, which decreased gradually, with an interference efficiency of 5.3% at 96 h of injection. After injection of dsGAD, the 72 h treatment showed the best interference efficiency (48%) at early-development stage, while 48 h after injection resulted in the lowest interference efficiency (23.1%). The best interference efficiency of the diapause stage was 82.1% at 96 h after injection, while no obvious interference effect at 24 h and 48 h of treatment. For diapause-terminated stage, the highest interference efficiency was 69.5% at 24 h, which gradually decreased, with the lowest value of 30.6% at 96 h. After injection of dsNOS, the interference efficiency reached the highest value of 67.9% at 96 h at the early-development stage, and no interference effect was recorded at 24 h and 48 h after injection. The interference efficiency of diapause stage reached the highest value of 42.8% at 48 h, and then decreased to the lowest value of 31.4% at 96 h. The interference efficiency of 96 h treatment was the best (81.8%) at the diapause-terminated stage, and that of 72 h was the lowest (50.4%).

### Effect of dsRNA treatment of cold tolerance genes on the hatching rate of *Calliptamus italicus* eggs

**Fig. 5:**
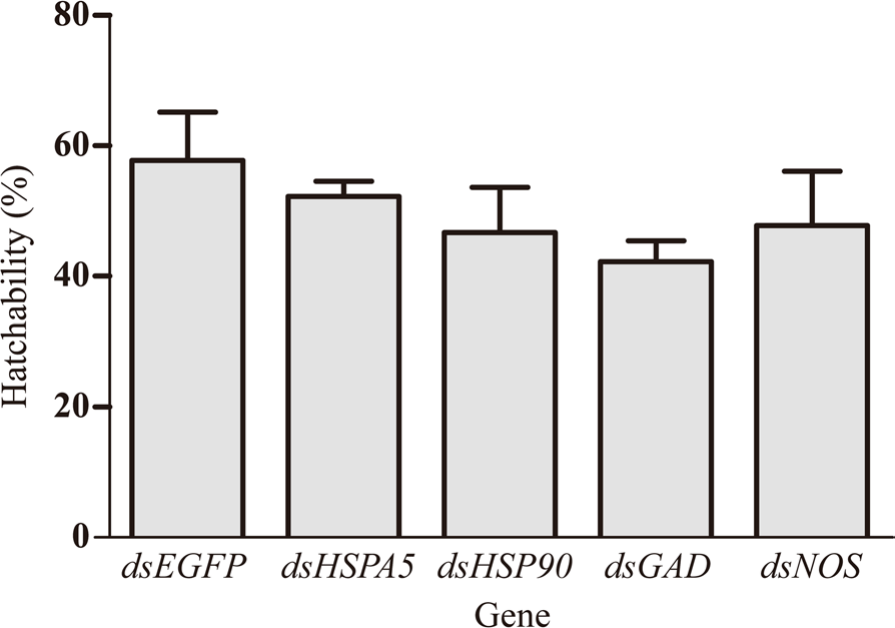
The effect of dsRNA of Hsp70, Hsp90, GAD and NOS treatment on cold tolerance of *C. italicus* egg hatchablilty

Compared with the control (57.78%), the hatching rate of *C. italicus* eggs decreased significantly after injection of dsHsp70, dsHsp90, dsGAD, and dsNOS (Fig. 5). The hatching rate after the injection of dsHsp70 was 52.22%, which decreased by 5.56%. The hatching rate with the injection of dsHsp90 was 46.67%, which decreased by 11.11%. The hatching rate after the injection of dsGAD was 43.33% with a decrease of 14.45%. The hatching rate after the injection of dsNOS was 47.78%, which decreased by 10%.

## Discussion

### Summary of transcriptome analysis

*C. italicus* is an important pest in the grassland of Xinjiang [21]. *C. italicus* eggs have strong cold resistance, but the molecular mechanism of cold resistance is still unclear [23, 38–40]. In this study, using RNA-Seq technology, nine transcriptomes of *C. italicus* eggs at three developmental stages were assembled at three different temperatures: constant low temperature acclimation (0 °C), outdoor natural low temperature acclimation in winter, and room temperature (27 °C). Compared with the control (27 °C), a large number of DEGs were screened after acclimation, with a greater number of down-regulated genes than that of the up-regulated genes, indicating that the negative regulation of these genes improved the cold tolerance in *C. italicus* eggs. Secondly, the number of DEGs in eggs under natural low-temperature acclimation was significantly higher than that of the under constant low-temperature acclimation, thus indicating that different acclimation modes can induce the expression of a large number of genes to alleviate the injury of low temperature. Whereas natural low-temperature acclimation induces the expression of more genes in response to low temperature, which can be speculated as the results of more sensitive response to fluctuating in *C. italicus* eggs. Wang et al. [41] found that low-temperature acclimation and simulated natural temperature acclimation significantly improved the survival rate of *Locusta migratoria* manilensis eggs. With a high survival rate for simulated natural temperature acclimation, the locusts eggs have better adaptability to natural temperature changes. After constant and natural low-temperature acclimation, 8 and 150 co-expressed genes were screened across the three different development stages in *C. italicus* eggs. However, only 2 and 22 co-expressed genes could be functional annotated, and they were preferentially enriched in pathways related to human diseases, thus their relevancy to cold tolerance needs to be further studied [42–44].

### Molecular mechanism of cold tolerance

The response of insects to temperature stress is a complex process, which requires the participation and regulation of multiple genes [45]. With the respect to the pathways involving DEGs, there were differences in the response mechanism of *C. italicus* eggs in low temperature under the two acclimation modes. Under constant low-temperature acclimation, metabolism pathway accounted for a large proportion of the significantly enriched pathways, in which a large number of DEGs were involved in sugar, lipid and amino acid metabolic pathways. Amino acid is an important osmoregulatory substance [46]. The accumulation of a large number of free amino acids in the hemolymph seemed to be an important physical and chemical feature for cold-tolerant insects in response to low temperature stress [47]. Ge et al. [38] also confirmed that amino acids, such as alanine, proline, tyrosine and phenylalanine, accumulated significantly in *C. italicus* eggs during overwintering. This allows us to speculate that pathways such as arginine and proline metabolism and beta-alanine metabolism play a key role in the response of *C. italicus* eggs to constant low-temperature acclimation. Under natural low-temperature acclimation, the organ system pathway accounted for a large proportion of the significantly enriched pathways, among which the digestive and the endocrine system can affect the growth, development and reproduction of insects [48]. For example, temperature stress can change the titer of juvenile hormone, resulting in the delay or failure of insect development. This abnormal development of insects led by temperature stress may be caused due to the abnormal endocrine system [49]. Thus, we speculated that the resistance to low temperature in *C. italicus* eggs could be achieved mainly by regulating the metabolic physiology under natural low-temperature acclimation. It is worth noting that DEGs were significantly enriched in the circadian rhythms pathway under both low-temperature acclimation modes, which is similar to the results of Parker et al. [50] Previous studies have shown that circadian rhythm related genes of insects are not only the molecular basis driving the output of their own physiological and behavioral circadian rhythms, but also related to the coping mechanism with temperature stress [51]. Still, it is unclear whether the changes in circadian rhythm genes directly affect the ability to tolerate cold. However, they can be promising candidates for explaining the metabolic changes during low-temperature acclimation [50].

Analysis of the significantly enriched, up-regulated pathways under the two low-temperature acclimation modes suggested the involvement of *GAD* gene under both constant low-temperature and natural low-temperature acclimation. Although GAD is the key enzyme functioning in catalysis of the decarboxylation of glutamate to produce γ-aminobutyric acid (GABA), it also accumulates abundantly in plants under various abiotic stresses [52], thus suggesting that GAD may be involved in regulating the normal physiological activities in the locust eggs at low temperature [53]. However, the expression of *GAD* in response to low temperature has not been reported. Also, the relationship between *GAD* gene and cold tolerance and its function needs to be further studied. *NOS* gene was also involved in cold acclimation in the two groups, although the pathways were different between groups. Thus, it can be speculated that the mechanism of *NOS* gene resisting low temperature may be different in the two low-temperature acclimation modes. Previous studies have shown that organisms can resist low temperature or other environmental stresses by up-regulating the expression of *NOS* [36, 54]. HSPs are closely related to the cold tolerance of insects [55, 56]. In the constant low-temperature acclimation group, the expression of *HspA5* gene was significantly up-regulated, while the expression of *Hsp90* gene was significantly up-regulated in the natural low-temperature acclimation group. Additionally, the expression of *HspA1_8* gene was significantly down-regulated due to low-temperature stress, thus implying that different *HSPs* genes showed different responses to different low temperature treatments, which is consistent with the results described in Zhang et al. [57].

The energy metabolism of insects would be inhibited at low temperature. Analysis of the significantly enriched, down-regulation pathways under the two acclimation modes found that the enzymes related to oxidative phosphorylation were significantly down-regulated in the constant low-temperature group, while the enzymes related to TCA cycle were significantly down-regulated in the natural low-temperature group. These results indicated that although the energy supply patterns for locust eggs were different in the two groups, they survived under low temperature conditions mainly through generation or consumption of less energy. This is in agreement with the mechanism that insects respond to low-temperature environmental pressure by inhibiting metabolic rate in winter [58]. Yan et al. [39] also proved that the level of respiration and metabolism of *C. italicus* eggs decreased significantly under low temperature condition.

In addition, AFPs that are related to cold tolerance in insects [59], have not been found under the two acclimation modes of locust eggs. One possible explanation is that only a few insect species were studied for the production of AFPs, which makes it difficult to identify AFP genes in newly studied species based on similarity annotation. On the other hand, the relationship between the levels of transcription and protein expression can be highly complex and often unequal. The analysis with the combination of transcriptome and proteome data often results in weak correlation between the levels of transcription and protein expression [60].

### Verification of cold tolerance gene

In this study, RNAi experiments were conducted by using the four selected genes. The results showed that the RNAi efficiency of different target genes was variable. Among the four genes injected with dsRNA, the highest interference efficiency of *dsHspA5* gene was about 80% at the three development stages, while the highest interference efficiency of *dsNOS* at the diapause stage was only 42.8%. *dsGAD* and *dsNOS* showed no interference effect at the early-development stage and the diapause stage at 24 h and 48 h after treatment. The results of Vatanparast et al. [61] also showed that after interfering on three important enzyme genes of *Helicoverpa armigera*, interference efficiency was found to be different (95.8, 97.7, and 74%).

Secondly, the optimal time for interference was also found to be different for various target genes. In the four periods for detection after injection of dsRNA, *dsHspA5* gene displayed the best interference effect at 72 h of early-development stage, 24 h of diapause stage and diapause-terminated stage, respectively. *dsHsp90* gene showed the best interference effect at 24 h of early-development stage and diapause stage, and 48 h of diapause-terminated stage respectively. *dsGAD* gene had the best interference effect at 72 h of the early-development stage, 96 h of the diapause stage and 24 h of the diapause-terminated stage, respectively. *dsNOS* gene produced the best interference effect at 96 h in the early-development stage and diapause-terminated stage, and 48 h in the diapause-terminated stage, respectively. Lin et al. [62] and Lü et al. [63] conducted interference experiments on *Henosepilachna vigintioctopunctata* and found that the larval mortality on the 3rd day after interfering with *HvIAPI* gene was 80%, while the same larval mortality could be achieved on the 9th day after interfering with *Hvlesswright* gene.

Thirdly, the interference effect was found to gradually weaken over time. For example, the expression level of *dsHspA5* gene decreased by about 80% after 24 h of interference in the diapause stage, but then increased gradually with the extension of interference time. This result indicated that the timeliness of RNA interference and the normal gene expression level will be restored after a certain period of time. Ji et al. [64] conducted an interference experiment on gene coding for cytochrome P450 reductase in *Spodoptera litura*. They found that the gene expression level decreased significantly, but the interference efficiency decreased gradually with the increase in interference time.

Fourthly, RNAi efficiency on the same gene could be different at different developmental stages. For example, the optimal interference efficiency of *dsGAD* gene was 48, 82 and 70% in the early-development, diapause, and diapause-terminated stage, respectively. This indicated that locust eggs had different sensitivity to dsRNA at different developmental stages. Hou [65] performed interference with the *BdCrzR* gene in *Bactrocera dorsalis* at different developmental periods. It was found that the silencing efficiency was 60% for larvae and 50% for adults.

Fifthly, compared with the control group, RNAi on target genes *HspA5*, *Hsp90*, *GAD* and *NOS* could not only reduce the expression of these genes, but also break the cold tolerance system of locust eggs. It tends to reduce their hatching rate after low temperature treatment due to the interference of the expression pattern of target genes, thus indicating that the four genes identified in this study play an important role in coping with low temperature stress in locust eggs.

In summary, the response to low-temperature stress in insects is a complex regulatory process involving multiple genes. RNAi study on a single gene cannot fully understand the mechanism of low-temperature stress tolerance in locust eggs. Locust eggs possess more than one copy of the four target genes, e.g., 17 *HSP70* genes have been found in locust eggs. Subsequent experiment of RNA interference can be performed on multiple gene members, to explore the role of specific genes in cold tolerance of locust eggs. Genes in specific pathways can also be interfered, with the aim to provide a basis for a comprehensive understanding of the mechanism of cold tolerance.

## Materials and methods

### Insects and treatments

In the early July 2019, female and male adults of *C. italicus* were captured in the Nanshan Experimental Station of Manas, Changji, Xinjiang Uygur Autonomous Region (43°54′ N, 86°7′ E; 1310 m), and were fed in an outdoor insect cage (1 m × 1 m × 1 m). Four plastic pots with the same diameter and a depth of 12 cm were placed on the ground in the cage. The pots were filled with sandy loam for *C. italicus* to lay eggs. The feeding density in the cage was 500 individuals/m^2^, and the ratio of female to male was kept at 1:1, aiming to obtain the social *C. italicus* [66]. The insects were fed daily with fresh *Artemisia frigida* and *Medicago sativa* until mating and spawning. The flower pots were replaced regularly on a daily basis, and the oocysts were collected by sieving the soil.

**Fig. 6:**
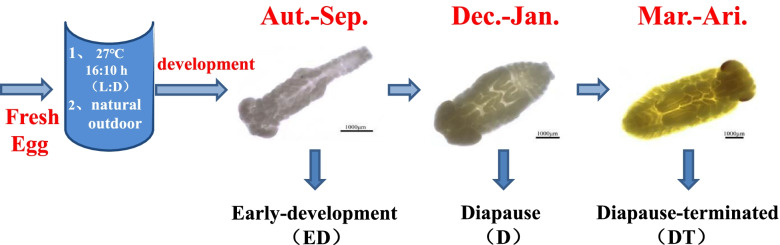
Egg development stage and sampling of *Calliptamus italicus*

After the oocysts were brought back to the laboratory, a portion of them were placed in a plastic box with a depth of about 5 cm that contained vermiculite(30 cm × 20 cm × 9.6 cm). The plastic box was sealed with a sealing film and pierced with small holes to maintain humidity and ventilation, and was then placed in an indoor intelligent artificial climate box. The remaining oocysts were placed in the soil about 5 cm deep under the natural outdoor conditions. In a pre-experiment, eggs in early-development stage were treated for 5 d, 10 d and 15 d at 0 °C and 4 °C. The results showed that the supercooling point at two temperatures after the treatment for 5 d was significantly different from that of the control group (27 °C) (*P*<0.05). The supercooling point at 0 °C after treatment for 10 d was significantly different from that of the control group (27 °C). The supercooling point at 0 °C after treatment for 15 days was significantly different from that of the control group (27 °C) (*P*<0.05). Since the strong tolerance of insects was seen in the diapauses stage [67], eggs treated at 0 °C for 15 d were set as experimental group I, eggs overwintering in outdoor natural conditions were set as experimental group II, and eggs at the same development stage in artificial incubator at 27 °C were set as control group. The eggs of groups I and II were separately sampled at the early-development, diapause and diapause-terminated stages, and 30 eggs in each group were sampled with three replicated groups. The development stages of overwintering eggs of *C. italicus* were divided according to the method described by Wang et al. [23] (Fig. 6). The difference in spawning time, temperature change in the year or storing temperature for eggs, can be the determining factors for the development time of *C. italicus* eggs [68]. Therefore, our experiment was carried out based on the development stages instead of the development time. The temperature of the artificial incubator was maintained at 27 ± 1 °C, while the humidity was set at 45% ± 5%, and the photoperiod was set as 14 L**:**10 D [69].

### Total RNA extraction, transcriptome sequencing and annotation

For each development stage of the experimental and the control group, 100 mg of *C. italicus* eggs was grounded with liquid nitrogen. The total RNA was extracted according to the instructions of TRIzol reagent. The quality of RNA was detected by 1% agarose gel electrophoresis, and the concentration and purity of RNA were evaluated by Ultramicro Biological Detector (Thermo, USA), and the qualified total RNA was further purified. Novogene (Tianjin, China) was commissioned to complete cDNA library construction and high-throughput sequencing. The sequencing platform was Illumina NovaSeq 6000, the sequencing read length was PE 150, and the sequencing method was “paired-end”, raw reads were obtained. Sequencing raw reads were preprocessed using Novogene’s internal Perl script, Clean reads were obtained by removing Adapter related, reads Containing N and Low quality reads. The obtained clean reads were assembled using Trinity (version: v2.4.0) software to obtain unigene. Finally, BLAST (version: V2.2.28 +, parameter: E-value < 10^− 5^) was used to compare unigene sequences with Nr, Nt, KOG and Swiss-Prot databases. Then use KAAS (version: R140224, parameter: e-value < 10^− 10^) to obtain the annotation information of unigene in KEGG. Use Blast2GO (version: B2G4PIPE_v2.5, parameter:e-value < 10^− 6^) to get the GO annotation information. The PFAM annotation information was obtained by using HMMSCAN (version: HMMER 3, parameter: e-value < 0.01) software.

### Analysis of differentially expressed genes (DEGs)

The FPKM method was used to calculate the expression of each annotated gene. The gene expression levels obtained were screened for the DEGs between the samples of constant low temperature acclimation group and the natural low temperature acclimation group with DESeq2 software. The screening threshold is |log2(FoldChange)| > 1 and *P*-value < 0.05. The smaller the *P*-value, the more significant the difference was in the gene expression. Finally, GOseq R and KOBAS software packages were used to analyze the GO and KEGG enrichment of DEGs.

### Validation of RNA-seq DEGs

RT-qPCR was used for the validation of gene expression, and several genes were randomly selected from the screened DEGs to verify the reliability of transcriptome data. Primers were designed by primer 5.0 software (Table S8), the cDNA synthesis mentioned in Section 4.2 was used as the template, and *β*-Actin gene was used as an internal reference. Fluorescence real-time quantitative PCR reaction was performed using SYBR® Premix Ex TaqTMgreen II kit. The reaction system (20 μL) consisted of: 1 μL of cDNA template, 1 μL of each gene-specific primer (0.2 μmol/L), 7 μL of ddH_2_O, and 10 μL of SYBR Green Supermix. The reaction program was: pre-denaturation at 95 °C for 10 min; 95 °C for 10 s, 58 °C for 15 s, 72 °C for 15 s, 40 cycles; 95 °C for 10 s, 65 °C for 60 s, and 97 °C for 1 s. The dissolution curves were then generated. Each group of *C. italicus* egg samples were repeated three times.

### Functional verification of cold tolerance genes by RNAi technology

Gene-specific primers with T7 promoter were designed (Table S9), and a 400–500 bp dsRNA was synthesized in the ORF region of the candidate cold tolerance gene using the TranscriptAidTM T7 kit. The concentration of dsRNA was measured by a microbiological detector and the integrity of dsRNA was detected by 1% agar gel electrophoresis. The qualified dsRNA was diluted in ddH2O and saved for later use.

The dsRNA was injected into the middle and upper yolk of the *C. italicus* eggs (27 °C) using a microinjector. The injection volume was 69 nL with the concentration of 1 μg/μL [70]. *C. italicus* eggs were injected with the same amount of dsEGFP which were used as control. After injection, a group of locust eggs were incubated in a 27 °C incubator for 24 h, 48 h, 72 h, and 96 h. The healthy and surviving *C. italicus* eggs were selected for each time period to extract total RNA and synthesize cDNA, which was detected by RT-qPCR. The reaction system and conditions were the same as those in Section 4.4. After low-temperature acclimation (0 °C/72 h), the other group was placed under the conditions of 27 ± 1 °C, 45 ± 5%, and L**:**D = 14**:**10 h, and the hatching rate was recorded. Each treatment was repeated three times, with 30 eggs for each repeat.

### Statistical analysis

The relative gene expression was calculated by 2^–△△*C*^_T_ method, and statistical analysis of the data was performed using SPSS 20.0 software. The interference efficiency of dsRNA at different time points was tested by independent sample *t*-test.

Hatching rate (%) = the number of hatched eggs/total locust eggs× 100%.

Significance level was tested using *P* < 0.05.

## Supplementary Information

**Additional file 1: Figure S1. A** Functional annotation of assembled sequences of DEGs of *C. italicus* egg at constant low-temperature acclimation (Z vs T) based on gene ontology (GO)categorization. Unigenes were annotated in three categories: biological process, cellular components, and molecular functions. **B** Functional annotation of assembled sequences of DEGs of *C. italicus* egg at natural low-temperature acclimation (N vs T) based on gene ontology (GO)categorization. Unigenes were annotated in three categories: biological process and molecular functions. **Figure S2. A** KEGG significant enrichment analysis for DEGs between early-development stage at constant low-temperature acclimation group (Z vs T) of *C. italicus* egg. **B** KEGG significant enrichment analysis for DEGs between diapause stage at constant low-temperature acclimation group (Z vs T) of *C. italicus* egg. **C** KEGG significant enrichment analysis for DEGs between diapause-terminated stage at constant low-temperature acclimation group (Z vs T) of *C. italicus* egg. **D** KEGG significant enrichment analysis for DEGs between early-development stage at natural low-temperature acclimation (N vs T) of *C. italicus* egg. **E** KEGG significant enrichment analysis for DEGs between diapause stage at natural low-temperature acclimation (N vs T)of *C. italicus* egg. **F** KEGG significant enrichment analysis for DEGs between diapause-terminated stage at natural low-temperature acclimation (N vs T) of *C. italicus* egg. **Table S1.** The information of DEGs. **Table S2.** Functional annotation of Significantly enriched GO at constant low-temperature acclimation (Z vs T). **Table S3.** Functional annotation of Significantly enriched GO at natural low-temperature acclimation(N vs T). **Table S4.** KEGG pathway enriched significantly at constant low-temperature acclimation (Z vs T). **Table S5.** KEGG pathway enriched significantly at natural low-temperature acclimation(N vs T). **Table S6.** qPCR verification results of transcriptomes. **Table S7.** Interference verification results of four cold tolerance genes. **Table S8.** Primers used for qPCR validation. **Table S9.** Primers used for dsRNA.

## Data Availability

All data generated or analyzed during this study are included in this article and its supplementary information files. We have uploaded the raw data of our transcriptome sequencing to NCBI BioProject repository: https://www.ncbi.nlm.nih.gov/bioproject/ PRJNA791798.

## Abbreviations

DEGs: Differentially expressed genes
RPKM: Read per kb per million
BLAST: Basic local alignment search tool
GO: Gene ontology
KEGG: Kyoto encyclopedia of genes and genomes
dsRNA: Double-stranded RNA
qPCR: Quantitative polymerase chain reaction
RNAi: RNA interference
HSPA5: Heat shock protein 70
HSP90: Heat shock protein 90
GAD: Glutamate decarboxylase
NOS: Nitric-oxide synthase
ALDH: Acetaldehyde dehydrogenase
TH: Tyrosine hydroxylase
HSPA1_8: Heat shock 70 kDa protein 1/8
CRYAB: Crystallin, alpha B
CAT, JUN, FASN, CALM: Calmodulin
PKA. SDH, PCK, FOXO3: Forkhead box protein O3

## References

[1] Sinclair BJ, Vernon P, Klok CJ, Chown SL (2003). Insects at low temperatures: an ecological perspective. Trends Ecol Evol.

[2] Ma CS, Ma G, Pincebourde S (2020). Survive a warming climate: insect responses to extreme high temperatures. Annu Rev Entomol.

[3] Lee RE (1989). Insect cold-hardiness: to freeze or not to freeze. BioScience.

[4] Vesala L, Salminen TS, Laiho A, Hoikkala A, Kankare M (2012). Cold tolerance and cold-induced modulation of gene expression in two Drosophila virilis group species with different distributions. Insect Mol Biol.

[5] Li NG, Toxopeus J, Moos M, Sørensen JG, Sinclair BJ (2020). A comparison of low temperature biology of Pieris rapae from Ontario, Canada, and Yakutia, far eastern Russia. Comp Biochem Phys A.

[6] Michaud MR, Denlinger DL (2004). Molecular modalities of insect cold survival: current understanding and future trends. Int Congress Ser.

[7] Cao KL, Wang FY, Hu M, Wuermaiti D, Roman J, Ji R (2018). Cold resistance and adaptation of *Pyrrhocoris apterus*. Acta Ecol Sin.

[8] Ganesan L, Fields PG, Jayas DS, Jian F (2021). Effects of developmental stage, cold acclimation and diet on the cold tolerance of three species of Cryptolestes (Coleoptera: Laemophloeidae). J Stored Prod Res.

[9] Colinet H, Rinehart JP, Yocum GD, Greenlee KJ (2018). Mechanisms underpinning the beneficial effects of fluctuating thermal regimes in insect cold tolerance. J Exp Biol.

[10] Carrington LB, Armijos MV, Lambrechts L, Barker CM, Scott TW (2013). Effects of fluctuating daily temperatures at critical thermal extremes on Aedes aegypti life-history traits. PLoS One.

[11] Shintani Y, Ishikawa Y (2007). Relationship between rapid cold-hardening and cold acclimation in the eggs of the yellow-spotted longicorn beetle. Psacothea hilaris J Insect Physiol.

[12] Wang BW, Hao X, Xu JY, Ma Y, Ma L (2019). Transcriptome-based analysis reveals a crucial role of *BxGPCR17454* in low temperature response of pine wood nematode (*Bursaphelenchus xylophilus*). Int J Mol Sci.

[13] Teets NM, Peyton JT, Colinet H, Renault D, Kelley JL, Kawarasaki Y, Lee RE, Denlinger DL (2012). Gene expression changes governing extreme dehydration tolerance in an Antarctic insect. PNAS..

[14] Moskalev A, Zhikrivetskaya S, Krasnov G, Shaposhnikov M, Proshkina E, Borisoglebsky D, Danilov A, Peregudova D, Sharapova I, Dobrovolskaya E, Solovev I, Zemskaya N, Shilova L, Snezhkina A, Kudryavtseva A (2015). A comparison of the transcriptome of *Drosophila melanogaster* in response to entomopathogenic fungus, ionizing radiation, starvation and cold shock. BMC Genomics.

[15] Li R, Wang YT, Jiang GF (2019). The transcriptome analysis of the bamboo grasshopper provides insights into hypothermic stress acclimation. Int J Biol Macromol.

[16] Dunning LT, Dennis AB, Sinclair BJ, Newcomb RD, Buckley TR (2014). Divergent transcriptional responses to low temperature among populations of alpine and lowland species of New Zealand stick insects *(Micrarchus)*. Mol Ecol.

[17] Yang CX, Ou D, Guo W, Lü J, Guo CF, Qiu BL, De Pan HP (2020). Novo assembly of the Asian Citrus psyllid Diaphorina citri (Hemiptera: Psyllidae) transcriptome across developmental stages. Int J Mol Sci.

[18] Camargo RA, Herai RH, Santos LN, Bento FMM, Lima JE, Marques-Souza H, Figueira A (2015). De Novo transcriptome assembly and analysis to identify potential gene targets for RNAi-mediated control of the tomato leafminer (*Tuta absoluta*). BMC Genomics.

[19] Huvenne H, Smagghe G (2010). Mechanisms of dsRNA uptake in insects and potential of RNAi for pest control: a review. J Insect Physiol.

[20] FAO Locust Watch Release, locust bulletin No.10, 2011. https://www.fao.org/ag/locusts-CCA/en/1010/1018/1075/.

[21] Zhang Q, Qiao Z, Xiong L, Bahatiyar D, Zhao Y, Dang HC, Zhang XS, Xiao HW (1995). Research on the biological characteristics of adult *Calliptamus italicus*. Xinjiang Agric Sci.

[22] Wang H, He XQ, Ji R (2010). Selection mechanisms of *Calliptamus italicus* on four different host plants. Chinese J Ecol.

[23] Wang XX, Yan MY, Zhang M, Yu CM, Yu F, Wang H, Hu HX, Yuan L, He L, Wang WL, Ji R, Ye XF (2019). Expression profiling of hemocyanin gene in the developmental process of eggs of *Calliptamus italicus* (Orthoptera: Catantopidae). Acta Entomol Sin.

[24] Maimatjang S, Mangura K (2020). Analysis and prediction of the characteristics of climate change in Xinjiang in the past 30 years. Agric Mach Agron.

[25] Zhang YM, Chen HY, Zhang WJ, Wang Y, Yu Y, Chen Q (2021). A transcriptome study of *Blattella germanica* in response to lowtemperature stress. Chin J Vector Biol Control.

[26] Tusong K, Guo XX, Meng SS, Liu XN, Ma J (2017). Comparative analysis of the transcriptome of the overwintering desert beetle *Microdera punctipennis*. Cryobiology.

[27] Yu SH, Yang P, Sun T, Qi Q, Wang XQ, Chen XM, Feng Y, Liu BW (2016). Transcriptomic and proteomic analyses on the supercooling ability and mining of antifreeze proteins of the Chinese white wax scale insect. Insect Sci.

[28] Mou D, Ma GL, Kazhuo CR, Li ZR, Zhong KJ, Xie JX, Li XL (2021). Comparative analysis of transcriptome of Caucasian clover *Trifolium ambiguum* Bieb. Under different cooling modes. Acta Agrestia Sinica.

[29] Kanehisa M, Goto S (2000). KEGG: Kyoto encyclopedia of genes and genomes. Nucleic Acids Res.

[30] Kanehisa M (2019). Toward understanding the origin and evolution of cellularorganisms. Protein Sci.

[31] Kanehisa M, Furumichi M, Sato Y, Ishiguro-Watanabe M, Tanabe M (2021). KEGG: integrating viruses and cellular organisms. Nucleic Acids Res.

[32] Kelty J, Ancevski A (2010). Rapid cold hardening in drosophila requires MAPK signaling. FASEB J.

[33] Teets NM, Yi SX, Lee RE, Denlinger DL (2013). Calcium signaling mediates cold sensing in insect tissues. Proc Natl Acad Sci.

[34] Zhou XR, Shan YM, Tan Y, Zhang ZR, Pang BP, Chiu J (2019). Comparative analysis of transcriptome responses to cold stress in *Galeruca daurica* (Coleoptera: Chrysomelidae). J Insect Sci.

[35] Niu DL, Zhao YE, Gong XJ, Yang R, Hu I, Zhang WY (2020). Stress response and silencing verification of heat shock proteins in Dermatophagoides farinae under temperature stress. Int J Biol Macromol.

[36] Jiang XB (2020). Preliminary study on diapause regulation by HSP70 and nitric oxide synthase in *Trichogramma dendrolimi.* MSc Thesis.

[37] Ran LY, Jiang CX, Yang QF, Wang HJ, Bai Ma TZ, Chen L, Kuang JK, Huang TT, Li Q (2020). Comparative transcriptome analysis of *Locusta migratoria tibetensis* Chen (Orthoptera: Oedipodidae) under high-and low-temperature stress. J Appl Entomol.

[38] Ge J, Ren JL, Zhao L (2014). Preliminary investigation changes in the free amino acids overwintering egg of *Calliptamus italicus* (L.) (Orthoptera: Catantopidae). *Xinjiang*. Agric Sci.

[39] Yan MY, Xu Y, Wang XX, Wang H, Ji R, Ye XF (2018). The response of respiratory metabolism in overwintering eggs of Italian locust *Calliptamus italicus* (Orthoptera: Catantopidae) to seasonal changes. J Plant Prot.

[40] Ren JL, Zhao L, Ge J, Tu XB (2021). Seasonal variation in cold hardiness and water content of *Calliptamus italicus* eggs. J. Plant Prot..

[41] Wang HS, Zhou CS, Guo W, Kang L (2006). Thermoperiodic acclimations enhance cold hardiness of the eggs of the migratory locust. Cryobiology.

[42] Chou RH, Wen HC, Liang WG, Lin SC, Yuan HW, Wu CW, Chang WWS (2011). Suppression of the invasion and migration of cancer cells by *SERPINB* family genes and their derived peptides. Oncol Rep.

[43] Li JC, Sun P, Huang T, He SD, Li LF, Xue G (2021). Individualized chemotherapy guided by the expression of *ERCC*1, *RRM*1, *TUBB*3, *TYMS* and *TOP2A* genes versus classic chemotherapy in the treatment of breast cancer: a comparative effectiveness study. Oncol Lett.

[44] Huijmans JGM, Schot R, Klerk JBCD, Williams M, Coo RFMD, Duran M, Mancini GMS, Verheijen FW, Slegtenhorst MV, Mancini GMS (2017). Molybdenum cofactor deficiency: identification of a patient with homozygote mutation in the *MOCS3* gene. Am J Med Genet A.

[45] Enriquez T, Colinet H (2019). Cold acclimation triggers major transcriptional changes in Drosophila suzukii. BMC Genomics.

[46] Doucet D, Walker VK, Qin W (2009). The bugs that came in from the cold: molecular adaptations to low temperatures in insects. Cell Mol Life Sci.

[47] Fields PG, Leuratlessard FF, Lavenseau L, Febvay G, Peypelut L, Bonnot G (1998). The effect of cold acclimation and deacclimation on cold tolerance, trehalose and free amino acid levels in sitophilus granarius and cryptolestes ferrugineus (coleoptera). J Insect Physiol.

[48] Hu SB (1989). Talking about the physiological basis of insects. Guangxi Plant Protection.

[49] David JR, Araripe LO, Chakir M, Legout H, Lemos B, Petavy G, Rohmer C, Joly D, Moreteau B (2005). Male sterility at extreme temperatures: a significant but neglected phenomenon for understanding Drosophila climatic adaptations. J Evol Biol.

[50] Parker DJ, Vesala L, Ritchie MG, Laiho A, Hoikkala A, Kankare M (2015). How consistent are the transcriptome changes associated with cold acclimation in two species of the Drosophila virilis group?. Heredity.

[51] Chu F, Qiu JF, Tao H, Li X, Shu MY, Liu HJ, SiMa Y, Xu S (2016). Impact of cyclical changes in temperature on circadian clock genes expression in Bombyx BmN cells. Arch Insect Biochem Physiol.

[52] Kennaway BM, Barry JS, Sriyani P, Alan WB (2003). Overexpression of glutamate decarboxylase in transgenic tobacco plants deters feeding by phytophagous insect larvae. J Chem Ecol.

[53] Wei D, Wang T, Dou W, Wang JJ (2014). Biochemical and molecular characteristics of glutamic decarboxylase from *Bactrocera dorsalis*. Sci Agric Sin.

[54] Sadekuzzaman M, Stanley D, Kim Y (2017). Nitric oxide mediates insect cellular immunity via phospholipase A_2_ activation. J Innate Immun.

[55] Sun M, Lu MX, Tang XT, Du YZ (2014). Characterization and expression of genes encoding three small heat shock proteins in *Sesamia inferens* (Lepidoptera: Noctuidae). Int J Mol Sci.

[56] Marteaux LED, McKinnon AH, Udaka H, Toxopeus J, Sinclair BJ (2017). Effects of cold-acclimation on gene expression in fall field cricket (*Gryllus pennsylvanicus*) ionoregulatory tissues. BMC Genomics.

[57] Zhang GJ, Storey JM, Storey KB (2011). Chaperone proteins and winter survival by a freeze tolerant insect. J Insect Physiol.

[58] Sinclair BJ (2015). Linking energetics and overwintering in temperate insects. J Therm Biol.

[59] Duman JG (2015). Animal ice-binding (antifreeze) proteins and glycolipids: an overview with emphasis on physiological function. J Exp Biol.

[60] Cui MM, Hu P, Wang T, Tao J, Zong S (2017). Differential transcriptome analysis reveals genes related to cold tolerance in seabuckthorn carpenter moth. Eogystia hippophaecolus PLoS ONE.

[61] Vatanparast M, Kazzazi M, Mirzaieasl A, Hosseininaveh V (2017). RNA interference-mediated knockdown of some involved in digestion and development of *Helicoverpa armigera*. B Entomol Res.

[62] Lin MJ, Guo MJ, Pan G, He SQ, Wu JH, Qiu BL, Pan HP (2021). RNAi-mediated silencing of *HvIAP1* affects survival and development of the 28-spotted ladybird, *Henosepilachna vigintioctopunctata*. J Environ Entomol.

[63] Lü J, Liu ZQ, Guo W, Guo MJ, Chen SM, Yang CX, Zhang YJ, Pan HP (2021). Oral delivery of dsHvlwr is a feasible method for managing the pest *Henosepilachna vigintioctopunctata* (Coleoptera: Coccinellidae). Insect Sci..

[64] Ji HY, Staehelin C, Jiang YP, Liu SW, Ma ZH, Su YJ, Zhang JE, Wang RL (2019). Tobacco cutworm (*Spodoptera Litura*) larvae silenced in the NADPH-cytochrome P450 reductase gene show increased susceptibility to Phoxim. Int J Mol Sci.

[65] Hou QL (2018). The physiological functions of Adipokinetic hormone and Corazonin signaling Systems in the Oriential Fruit fly, *Bactrocera dorsalis* (Hendel) (Diptera: Tephritidae).

[66] Zhang Y (2011). Study on ecotype and biological characteristics of *Calliptamus italicus* (L.) (Orthoptera: Catantopidae) MSc Thesis.

[67] MacRae TH (2010). Gene expression, metabolic regulation and stress tolerance during diapause. Cell Mol Life Sci.

[68] Ren JL, Zhao L, Ge J (2015). Embryonic development of diapausing eggs in *Calliptamus italicus* (L.)(Orthoptera: Catantopidae). Acta Entomol Sin.

[69] Ren JL, Zhao L, Gong A (2015). Effects of the temperature and soil moisture on postdiapause development and survival of *Calliptamus italicus* L. eggs. J Xinjiang Agric Univ.

[70] Ma GL (2014). Effects of Hemocyanin’s genetic on embryonic development in the migratory locust. MSc Thesis.

