# Supplementary material for: Transcriptome analysis of the response to low temperature acclimation in Calliptamus italicus eggs
Source: BMC Genomics. 2022 Jul 1;23:482. doi: 10.1186/s12864-022-08705-3 (PMC9248191; doi:10.1186/s12864-022-08705-3)
Supplement: Supplementary file 1 — Additional file 1: Figure S1. A Functional annotation of assembled sequences of DEGs of C. italicus egg at constant low-temperature acclimation (Z vs T) based on gene ontology (GO)categorization. Unigenes were annotated in three categories: biological process, cellular components, and molecular functions. B Functional annotation of assembled sequences of DEGs of C. italicus egg at natural low-temperature acclimation (N vs T) based on gene ontology (GO)categorization. Unigenes were annotated in three categories: biological process and molecular functions. Figure S2. A KEGG significant enrichment analysis for DEGs between early-development stage at constant low-temperature acclimation group (Z vs T) of C. italicus egg. B KEGG significant enrichment analysis for DEGs between diapause stage at constant low-temperature acclimation group (Z vs T) of C. italicus egg. C KEGG significant enrichment analysis for DEGs between diapause-terminated stage at constant low-temperature acclimation group (Z vs T) of C. italicus egg. D KEGG significant enrichment analysis for DEGs between early-development stage at natural low-temperature acclimation (N vs T) of C. italicus egg. E KEGG significant enrichment analysis for DEGs between diapause stage at natural low-temperature acclimation (N vs T)of C. italicus egg. F KEGG significant enrichment analysis for DEGs between diapause-terminated stage at natural low-temperature acclimation (N vs T) of C. italicus egg. Table S1. The information of DEGs. Table S2. Functional annotation of Significantly enriched GO at constant low-temperature acclimation (Z vs T). Table S3. Functional annotation of Significantly enriched GO at natural low-temperature acclimation(N vs T). Table S4. KEGG pathway enriched significantly at constant low-temperature acclimation (Z vs T). Table S5. KEGG pathway enriched significantly at natural low-temperature acclimation(N vs T). Table S6. qPCR verification results of transcriptomes. Table S7. Interference verificatio [file 12864_2022_8705_MOESM1_ESM.zip › Supplementary Information/Table S8 Primers used for qPCR validation..docx]

Table S1: specific information of qPCR primers

| Primers | Primer sequences (5′-3′) | Amplified length (bp) |
| --- | --- | --- |
| qHSPA5-F | GGAGAACAGGACACAGATGC | 211 |
| qHSPA5-R | CAAACAGCGATGCCATTACAC |  |
| qHSP90A-F | CCGAAGCCGACAAGAATG | 147 |
| qHSP90A-R | CAATGCCCAGACCCAACT |  |
| qGAD-F | TGTGGCAGAAGAGCAGATGT | 197 |
| qGAD-R | GATGGCGGAATGTAGCAGAAG |  |
| qNOS1-F | TTCACCGCTGCTCCTTTCA | 108 |
| qNOS1-R | CATTCTTGTCGCTACCGTCTC |  |
| qCYP4-F | CGCCGCCTAGATTCTTCAG | 129 |
| qCYP4-R | TATCCAGCATCCAGTCCAACAG |  |
| qPER-F | TGGCTCATCTGACGGAACAT | 157 |
| qPER-R | GGCACGCAACTTCTGTAGG |  |
| qCTSC-F | CGCTGGAACGAATGCCTTG | 180 |
| qCTSC-R | CGTACTTGCCGAACCACATC |  |
| qSDH1-F | GCACTGGAGGAAGCATACAC | 110 |
| qSDH1-R | CGGTAGCACATTCTCGTTCAT |  |
| qBOI-F | CGCCTCGTGTCTTCTCTGT | 140 |
| qBOI-R | ATGCCCAAGTGCCCAAC |  |
| qCAT-F | CCCGAGTTGTTAGAAAGGAGAG | 164 |
| qCAT-R | GCTGGCTGTATGGCTGTCA |  |
| qJUN-F | GTCAACCACCTCAAGCGTAG | 106 |
| qJUN-R | CATCCATCCCGTTCGTCAC |  |
| qVRI-F | TAACAGCCTGCCGCACAAG | 167 |
| qVRI-R | CCAAGGAGATGCCAGAGTCG |  |
| qDDX5-F | TGGATGAGGCTGACCGTATG | 104 |
| qDDX5-R | GTAGCAGACCACAGGAGAGT |  |
| qdnaK-F | GAAAGATGAGACCGACCCTG | 137 |
| qdnaK-R | CACTGCTGCCACTACTTCC |  |
| qCCNH-F | CATCTGGTAGGGAACATCTCTCT | 207 |
| qCCNH-R | CATCATCGTCGTCATCGTCATCA |  |
| β-actin-F | AAGGCATCAGGGTGTGATGG | 177 |
| β-actin-R | GCCACCCTAAGCTCGTTGTA |  |
