# Supplementary material for: Transcriptome analysis of the response to low temperature acclimation in Calliptamus italicus eggs
Source: BMC Genomics. 2022 Jul 1;23:482. doi: 10.1186/s12864-022-08705-3 (PMC9248191; doi:10.1186/s12864-022-08705-3)
Supplement: Supplementary file 1 — Additional file 1: Figure S1. A Functional annotation of assembled sequences of DEGs of C. italicus egg at constant low-temperature acclimation (Z vs T) based on gene ontology (GO)categorization. Unigenes were annotated in three categories: biological process, cellular components, and molecular functions. B Functional annotation of assembled sequences of DEGs of C. italicus egg at natural low-temperature acclimation (N vs T) based on gene ontology (GO)categorization. Unigenes were annotated in three categories: biological process and molecular functions. Figure S2. A KEGG significant enrichment analysis for DEGs between early-development stage at constant low-temperature acclimation group (Z vs T) of C. italicus egg. B KEGG significant enrichment analysis for DEGs between diapause stage at constant low-temperature acclimation group (Z vs T) of C. italicus egg. C KEGG significant enrichment analysis for DEGs between diapause-terminated stage at constant low-temperature acclimation group (Z vs T) of C. italicus egg. D KEGG significant enrichment analysis for DEGs between early-development stage at natural low-temperature acclimation (N vs T) of C. italicus egg. E KEGG significant enrichment analysis for DEGs between diapause stage at natural low-temperature acclimation (N vs T)of C. italicus egg. F KEGG significant enrichment analysis for DEGs between diapause-terminated stage at natural low-temperature acclimation (N vs T) of C. italicus egg. Table S1. The information of DEGs. Table S2. Functional annotation of Significantly enriched GO at constant low-temperature acclimation (Z vs T). Table S3. Functional annotation of Significantly enriched GO at natural low-temperature acclimation(N vs T). Table S4. KEGG pathway enriched significantly at constant low-temperature acclimation (Z vs T). Table S5. KEGG pathway enriched significantly at natural low-temperature acclimation(N vs T). Table S6. qPCR verification results of transcriptomes. Table S7. Interference verificatio [file 12864_2022_8705_MOESM1_ESM.zip › Supplementary Information/Table S9 Primers used for dsRNA..docx]

Table S2: specific information of dsRNA primers.

| Primers | Primer sequences (5′-3′) | Amplified length (bp) |
| --- | --- | --- |
| HSPA5_F | **TAATACGACTCACTATAGGG**GTCTTGTTGTGATGAGGAT | 454 |
| HSPA5_R | **TAATACGACTCACTATAGGG**CACAGGCTTCATAGTAGA |  |
| HSP90_F | **TAATACGACTCACTATAGGG**CTCTGCTGTTCATTCCT | 456 |
| HSP90_R | **TAATACGACTCACTATAGGG**TGTTTCTGGTTCTCTTTC |  |
| GAD_F | **TAATACGACTCACTATAGGG**AGTGCTCAATACCTATTCCA | 493 |
| GAD_R | **TAATACGACTCACTATAGGG**TCCCTTCCAAGTCTCTCA |  |
| NOS_F | **TAATACGACTCACTATAGGG**AGCCTCTTATTATGGTTGGA | 498 |
| NOS_R | **TAATACGACTCACTATAGGG**TTCTCACCTCTGCTGTTG |  |
| EGFP-F | **TAATACGACTCACTATAGGG**CACCTACGGCAAGCTGACCCTGAA | 563 |
| EGFP-R | **TAATACGACTCACTATAGGG**TGGGTGCTCAGGTAGTGGTTGTCG |  |
