# Supplementary figures and images for: Transcriptome analysis of the response to low temperature acclimation in Calliptamus italicus eggs
Source: BMC Genomics. 2022 Jul 1;23:482. doi: 10.1186/s12864-022-08705-3 (PMC9248191; doi:10.1186/s12864-022-08705-3)

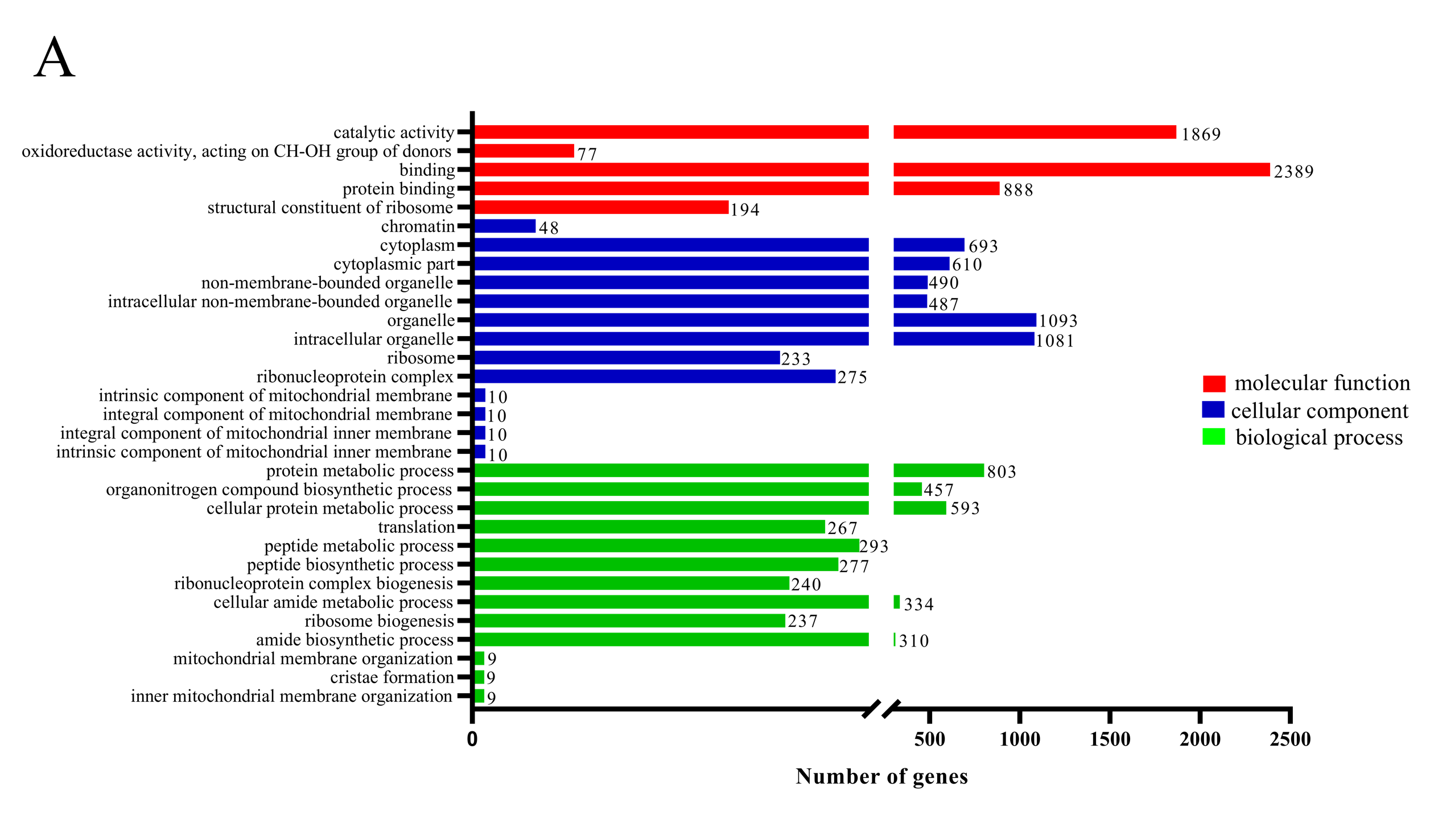

Supplement: Supplementary file 1 — Additional file 1: Figure S1. A Functional annotation of assembled sequences of DEGs of C. italicus egg at constant low-temperature acclimation (Z vs T) based on gene ontology (GO)categorization. Unigenes were annotated in three categories: biological process, cellular components, and molecular functions. B Functional annotation of assembled sequences of DEGs of C. italicus egg at natural low-temperature acclimation (N vs T) based on gene ontology (GO)categorization. Unigenes were annotated in three categories: biological process and molecular functions. Figure S2. A KEGG significant enrichment analysis for DEGs between early-development stage at constant low-temperature acclimation group (Z vs T) of C. italicus egg. B KEGG significant enrichment analysis for DEGs between diapause stage at constant low-temperature acclimation group (Z vs T) of C. italicus egg. C KEGG significant enrichment analysis for DEGs between diapause-terminated stage at constant low-temperature acclimation group (Z vs T) of C. italicus egg. D KEGG significant enrichment analysis for DEGs between early-development stage at natural low-temperature acclimation (N vs T) of C. italicus egg. E KEGG significant enrichment analysis for DEGs between diapause stage at natural low-temperature acclimation (N vs T)of C. italicus egg. F KEGG significant enrichment analysis for DEGs between diapause-terminated stage at natural low-temperature acclimation (N vs T) of C. italicus egg. Table S1. The information of DEGs. Table S2. Functional annotation of Significantly enriched GO at constant low-temperature acclimation (Z vs T). Table S3. Functional annotation of Significantly enriched GO at natural low-temperature acclimation(N vs T). Table S4. KEGG pathway enriched significantly at constant low-temperature acclimation (Z vs T). Table S5. KEGG pathway enriched significantly at natural low-temperature acclimation(N vs T). Table S6. qPCR verification results of transcriptomes. Table S7. Interference verificatio [file 12864_2022_8705_MOESM1_ESM.zip › Supplementary Information/Figure S1,A Functional annotation of assembled sequences of DEGs of C. italicus egg at constant low-temperature acclimation (Z vs T) based on gene ontology (GO)categorization..tif]

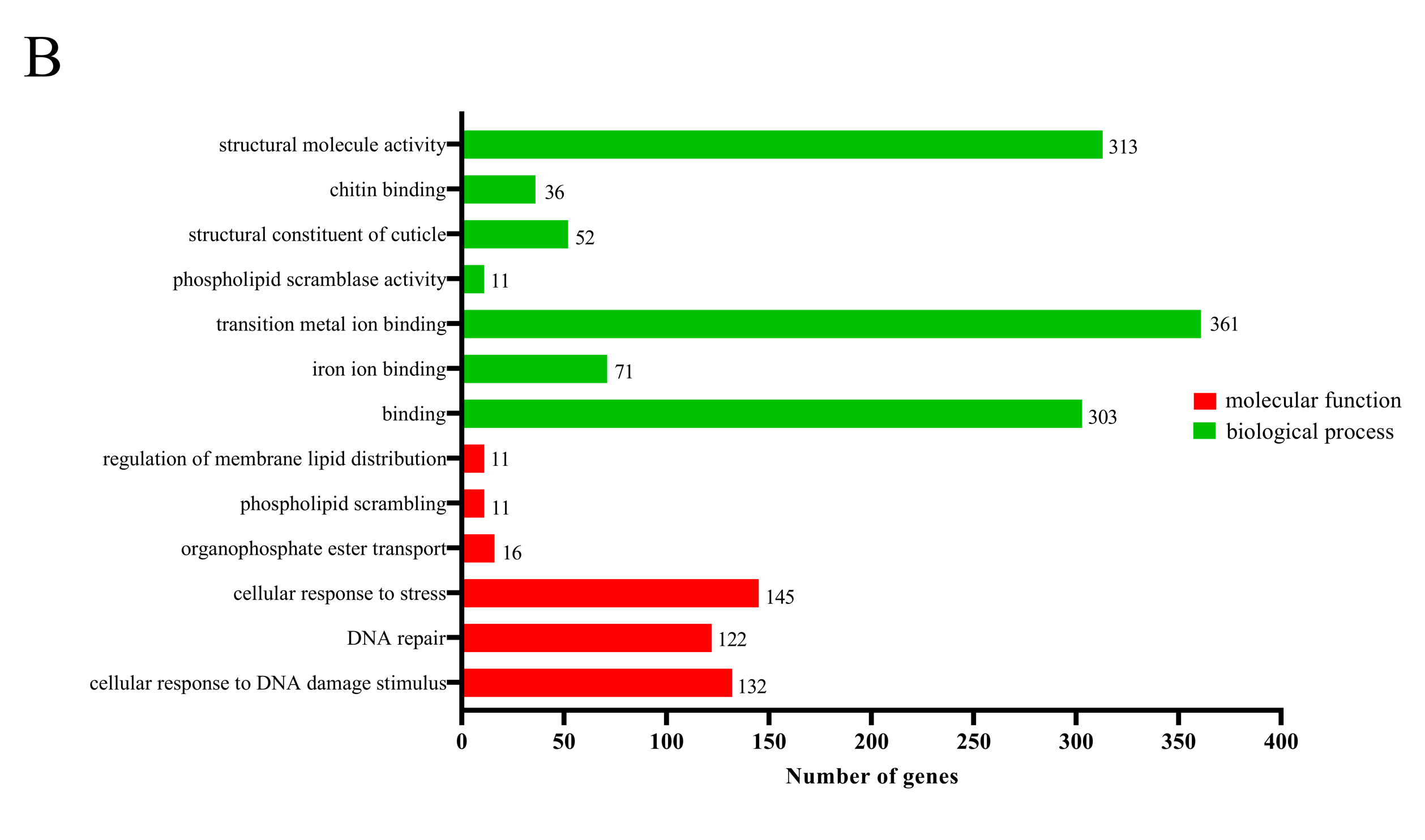

Supplement: Supplementary file 1 — Additional file 1: Figure S1. A Functional annotation of assembled sequences of DEGs of C. italicus egg at constant low-temperature acclimation (Z vs T) based on gene ontology (GO)categorization. Unigenes were annotated in three categories: biological process, cellular components, and molecular functions. B Functional annotation of assembled sequences of DEGs of C. italicus egg at natural low-temperature acclimation (N vs T) based on gene ontology (GO)categorization. Unigenes were annotated in three categories: biological process and molecular functions. Figure S2. A KEGG significant enrichment analysis for DEGs between early-development stage at constant low-temperature acclimation group (Z vs T) of C. italicus egg. B KEGG significant enrichment analysis for DEGs between diapause stage at constant low-temperature acclimation group (Z vs T) of C. italicus egg. C KEGG significant enrichment analysis for DEGs between diapause-terminated stage at constant low-temperature acclimation group (Z vs T) of C. italicus egg. D KEGG significant enrichment analysis for DEGs between early-development stage at natural low-temperature acclimation (N vs T) of C. italicus egg. E KEGG significant enrichment analysis for DEGs between diapause stage at natural low-temperature acclimation (N vs T)of C. italicus egg. F KEGG significant enrichment analysis for DEGs between diapause-terminated stage at natural low-temperature acclimation (N vs T) of C. italicus egg. Table S1. The information of DEGs. Table S2. Functional annotation of Significantly enriched GO at constant low-temperature acclimation (Z vs T). Table S3. Functional annotation of Significantly enriched GO at natural low-temperature acclimation(N vs T). Table S4. KEGG pathway enriched significantly at constant low-temperature acclimation (Z vs T). Table S5. KEGG pathway enriched significantly at natural low-temperature acclimation(N vs T). Table S6. qPCR verification results of transcriptomes. Table S7. Interference verificatio [file 12864_2022_8705_MOESM1_ESM.zip › Supplementary Information/Figure S1,B Functional annotation of assembled sequences of DEGs of C. italicus egg at natural low-temperature acclimation (N vs T) based on gene ontology (GO)categorization..tif]

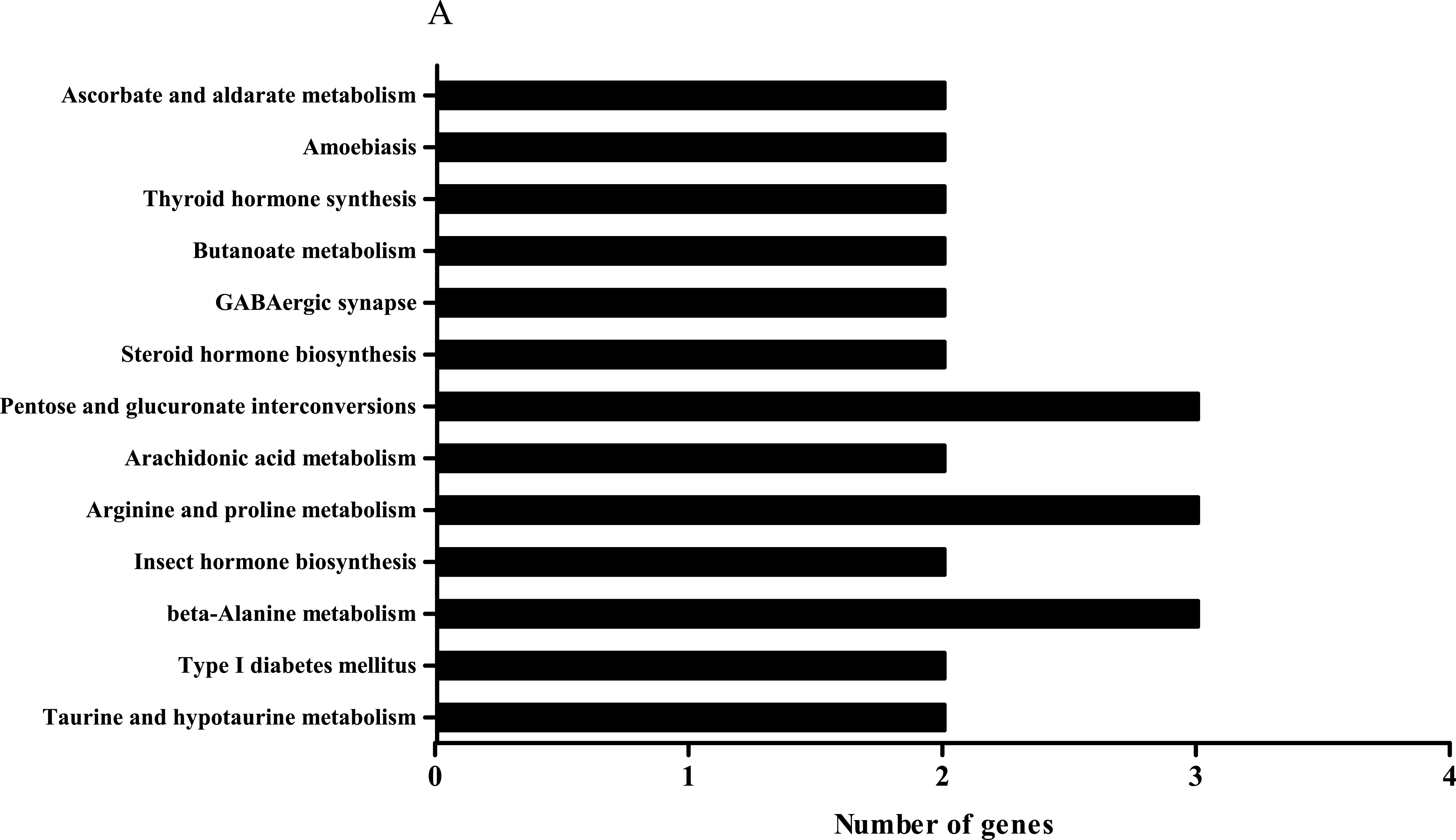

Supplement: Supplementary file 1 — Additional file 1: Figure S1. A Functional annotation of assembled sequences of DEGs of C. italicus egg at constant low-temperature acclimation (Z vs T) based on gene ontology (GO)categorization. Unigenes were annotated in three categories: biological process, cellular components, and molecular functions. B Functional annotation of assembled sequences of DEGs of C. italicus egg at natural low-temperature acclimation (N vs T) based on gene ontology (GO)categorization. Unigenes were annotated in three categories: biological process and molecular functions. Figure S2. A KEGG significant enrichment analysis for DEGs between early-development stage at constant low-temperature acclimation group (Z vs T) of C. italicus egg. B KEGG significant enrichment analysis for DEGs between diapause stage at constant low-temperature acclimation group (Z vs T) of C. italicus egg. C KEGG significant enrichment analysis for DEGs between diapause-terminated stage at constant low-temperature acclimation group (Z vs T) of C. italicus egg. D KEGG significant enrichment analysis for DEGs between early-development stage at natural low-temperature acclimation (N vs T) of C. italicus egg. E KEGG significant enrichment analysis for DEGs between diapause stage at natural low-temperature acclimation (N vs T)of C. italicus egg. F KEGG significant enrichment analysis for DEGs between diapause-terminated stage at natural low-temperature acclimation (N vs T) of C. italicus egg. Table S1. The information of DEGs. Table S2. Functional annotation of Significantly enriched GO at constant low-temperature acclimation (Z vs T). Table S3. Functional annotation of Significantly enriched GO at natural low-temperature acclimation(N vs T). Table S4. KEGG pathway enriched significantly at constant low-temperature acclimation (Z vs T). Table S5. KEGG pathway enriched significantly at natural low-temperature acclimation(N vs T). Table S6. qPCR verification results of transcriptomes. Table S7. Interference verificatio [file 12864_2022_8705_MOESM1_ESM.zip › Supplementary Information/Figure S2,A KEGG significant enrichment analysis for DEGs between early-development stage at constant low-temperature acclimation group (Z vs T) of C. italicus egg.jpg]

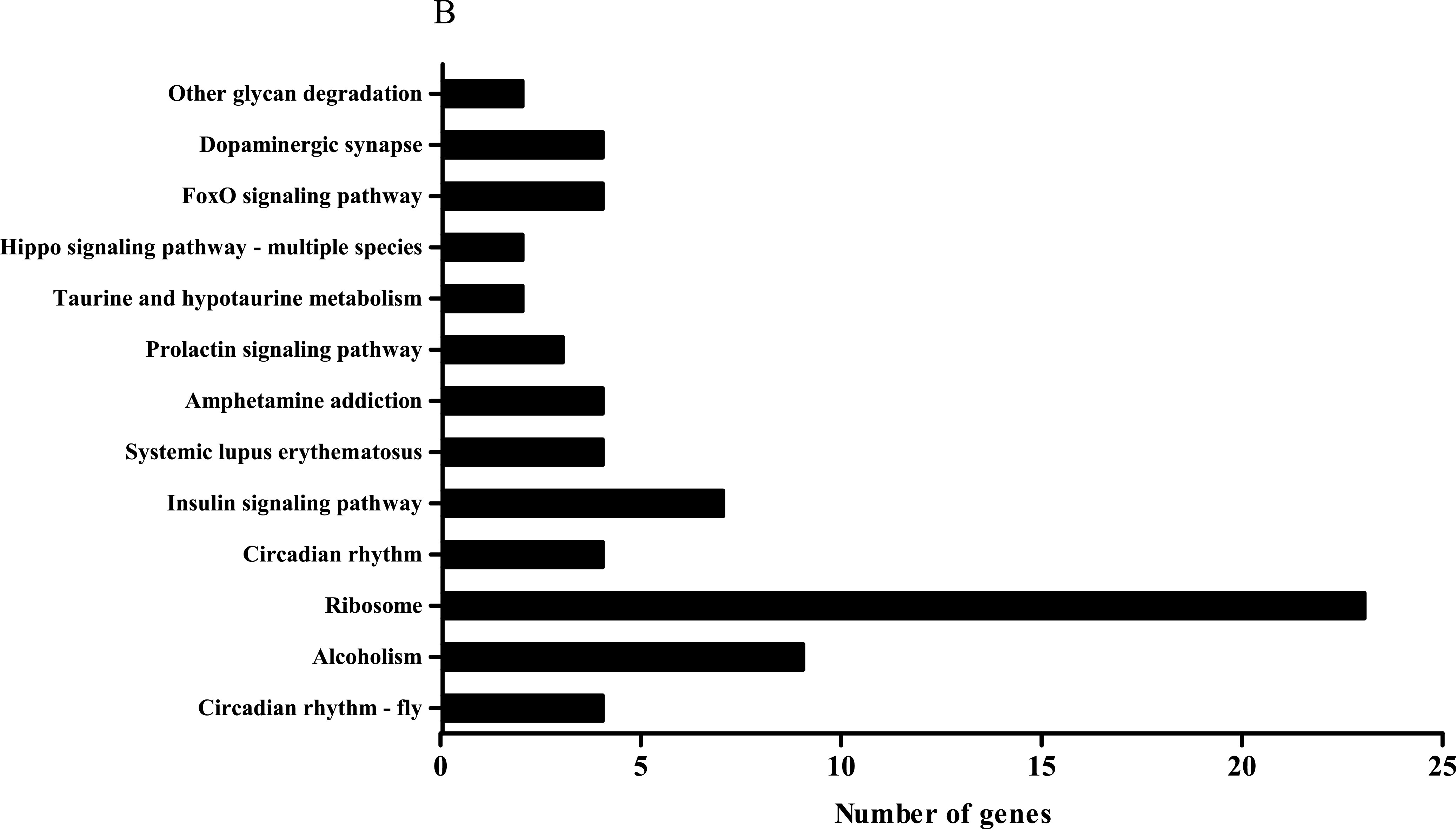

Supplement: Supplementary file 1 — Additional file 1: Figure S1. A Functional annotation of assembled sequences of DEGs of C. italicus egg at constant low-temperature acclimation (Z vs T) based on gene ontology (GO)categorization. Unigenes were annotated in three categories: biological process, cellular components, and molecular functions. B Functional annotation of assembled sequences of DEGs of C. italicus egg at natural low-temperature acclimation (N vs T) based on gene ontology (GO)categorization. Unigenes were annotated in three categories: biological process and molecular functions. Figure S2. A KEGG significant enrichment analysis for DEGs between early-development stage at constant low-temperature acclimation group (Z vs T) of C. italicus egg. B KEGG significant enrichment analysis for DEGs between diapause stage at constant low-temperature acclimation group (Z vs T) of C. italicus egg. C KEGG significant enrichment analysis for DEGs between diapause-terminated stage at constant low-temperature acclimation group (Z vs T) of C. italicus egg. D KEGG significant enrichment analysis for DEGs between early-development stage at natural low-temperature acclimation (N vs T) of C. italicus egg. E KEGG significant enrichment analysis for DEGs between diapause stage at natural low-temperature acclimation (N vs T)of C. italicus egg. F KEGG significant enrichment analysis for DEGs between diapause-terminated stage at natural low-temperature acclimation (N vs T) of C. italicus egg. Table S1. The information of DEGs. Table S2. Functional annotation of Significantly enriched GO at constant low-temperature acclimation (Z vs T). Table S3. Functional annotation of Significantly enriched GO at natural low-temperature acclimation(N vs T). Table S4. KEGG pathway enriched significantly at constant low-temperature acclimation (Z vs T). Table S5. KEGG pathway enriched significantly at natural low-temperature acclimation(N vs T). Table S6. qPCR verification results of transcriptomes. Table S7. Interference verificatio [file 12864_2022_8705_MOESM1_ESM.zip › Supplementary Information/Figure S2,B KEGG significant enrichment analysis for DEGs between diapause stage at constant low-temperature acclimation group (Z vs T) of C. italicus egg.jpg]

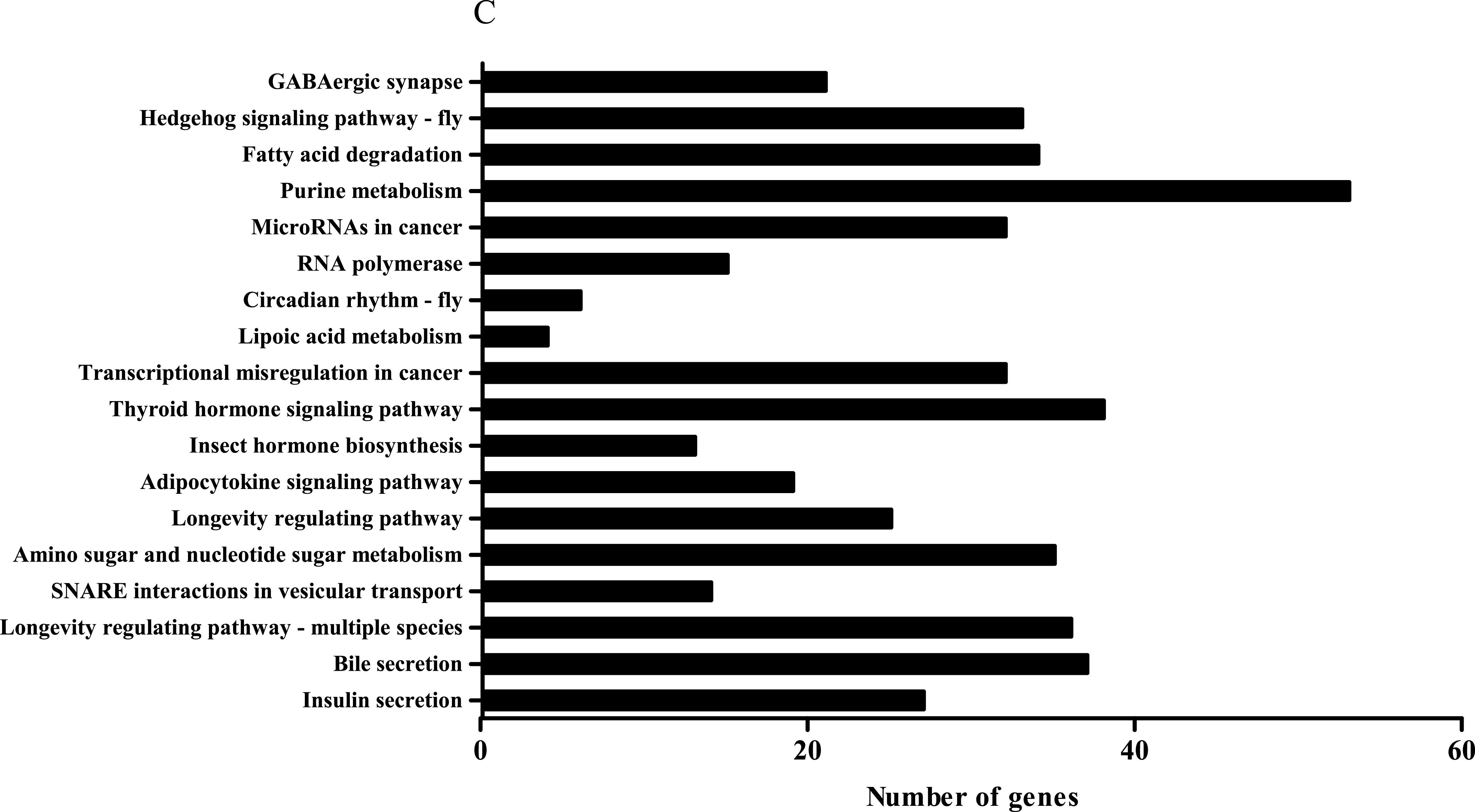

Supplement: Supplementary file 1 — Additional file 1: Figure S1. A Functional annotation of assembled sequences of DEGs of C. italicus egg at constant low-temperature acclimation (Z vs T) based on gene ontology (GO)categorization. Unigenes were annotated in three categories: biological process, cellular components, and molecular functions. B Functional annotation of assembled sequences of DEGs of C. italicus egg at natural low-temperature acclimation (N vs T) based on gene ontology (GO)categorization. Unigenes were annotated in three categories: biological process and molecular functions. Figure S2. A KEGG significant enrichment analysis for DEGs between early-development stage at constant low-temperature acclimation group (Z vs T) of C. italicus egg. B KEGG significant enrichment analysis for DEGs between diapause stage at constant low-temperature acclimation group (Z vs T) of C. italicus egg. C KEGG significant enrichment analysis for DEGs between diapause-terminated stage at constant low-temperature acclimation group (Z vs T) of C. italicus egg. D KEGG significant enrichment analysis for DEGs between early-development stage at natural low-temperature acclimation (N vs T) of C. italicus egg. E KEGG significant enrichment analysis for DEGs between diapause stage at natural low-temperature acclimation (N vs T)of C. italicus egg. F KEGG significant enrichment analysis for DEGs between diapause-terminated stage at natural low-temperature acclimation (N vs T) of C. italicus egg. Table S1. The information of DEGs. Table S2. Functional annotation of Significantly enriched GO at constant low-temperature acclimation (Z vs T). Table S3. Functional annotation of Significantly enriched GO at natural low-temperature acclimation(N vs T). Table S4. KEGG pathway enriched significantly at constant low-temperature acclimation (Z vs T). Table S5. KEGG pathway enriched significantly at natural low-temperature acclimation(N vs T). Table S6. qPCR verification results of transcriptomes. Table S7. Interference verificatio [file 12864_2022_8705_MOESM1_ESM.zip › Supplementary Information/Figure S2,C KEGG significant enrichment analysis for DEGs between diapause-terminated stage at constant low-temperature acclimation group (Z vs T) of C. italicus egg.jpg]

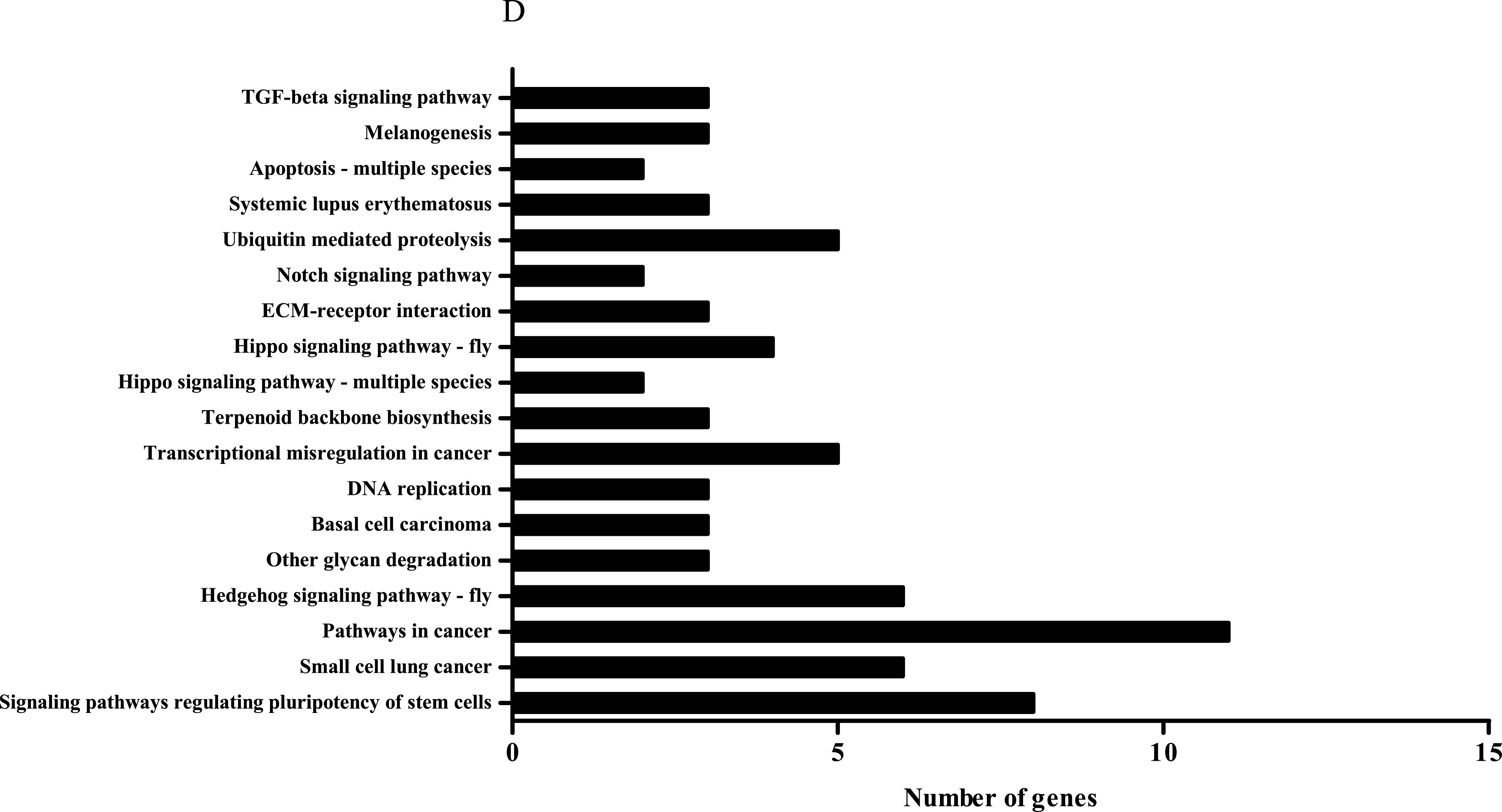

Supplement: Supplementary file 1 — Additional file 1: Figure S1. A Functional annotation of assembled sequences of DEGs of C. italicus egg at constant low-temperature acclimation (Z vs T) based on gene ontology (GO)categorization. Unigenes were annotated in three categories: biological process, cellular components, and molecular functions. B Functional annotation of assembled sequences of DEGs of C. italicus egg at natural low-temperature acclimation (N vs T) based on gene ontology (GO)categorization. Unigenes were annotated in three categories: biological process and molecular functions. Figure S2. A KEGG significant enrichment analysis for DEGs between early-development stage at constant low-temperature acclimation group (Z vs T) of C. italicus egg. B KEGG significant enrichment analysis for DEGs between diapause stage at constant low-temperature acclimation group (Z vs T) of C. italicus egg. C KEGG significant enrichment analysis for DEGs between diapause-terminated stage at constant low-temperature acclimation group (Z vs T) of C. italicus egg. D KEGG significant enrichment analysis for DEGs between early-development stage at natural low-temperature acclimation (N vs T) of C. italicus egg. E KEGG significant enrichment analysis for DEGs between diapause stage at natural low-temperature acclimation (N vs T)of C. italicus egg. F KEGG significant enrichment analysis for DEGs between diapause-terminated stage at natural low-temperature acclimation (N vs T) of C. italicus egg. Table S1. The information of DEGs. Table S2. Functional annotation of Significantly enriched GO at constant low-temperature acclimation (Z vs T). Table S3. Functional annotation of Significantly enriched GO at natural low-temperature acclimation(N vs T). Table S4. KEGG pathway enriched significantly at constant low-temperature acclimation (Z vs T). Table S5. KEGG pathway enriched significantly at natural low-temperature acclimation(N vs T). Table S6. qPCR verification results of transcriptomes. Table S7. Interference verificatio [file 12864_2022_8705_MOESM1_ESM.zip › Supplementary Information/Figure S2,D KEGG significant enrichment analysis for DEGs between early-development stage at natural low-temperature acclimation (N vs T) of C. italicus egg.jpg]

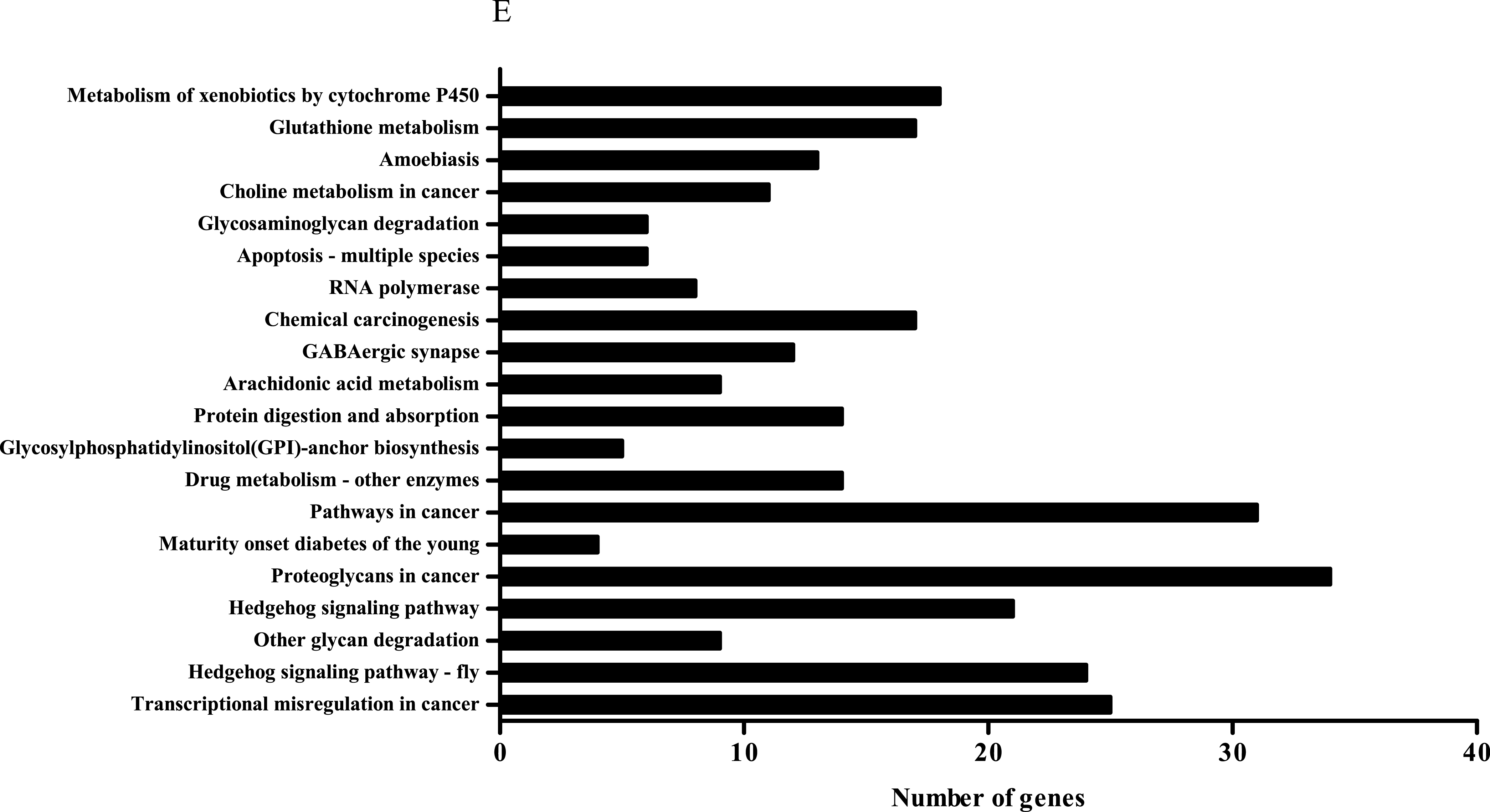

Supplement: Supplementary file 1 — Additional file 1: Figure S1. A Functional annotation of assembled sequences of DEGs of C. italicus egg at constant low-temperature acclimation (Z vs T) based on gene ontology (GO)categorization. Unigenes were annotated in three categories: biological process, cellular components, and molecular functions. B Functional annotation of assembled sequences of DEGs of C. italicus egg at natural low-temperature acclimation (N vs T) based on gene ontology (GO)categorization. Unigenes were annotated in three categories: biological process and molecular functions. Figure S2. A KEGG significant enrichment analysis for DEGs between early-development stage at constant low-temperature acclimation group (Z vs T) of C. italicus egg. B KEGG significant enrichment analysis for DEGs between diapause stage at constant low-temperature acclimation group (Z vs T) of C. italicus egg. C KEGG significant enrichment analysis for DEGs between diapause-terminated stage at constant low-temperature acclimation group (Z vs T) of C. italicus egg. D KEGG significant enrichment analysis for DEGs between early-development stage at natural low-temperature acclimation (N vs T) of C. italicus egg. E KEGG significant enrichment analysis for DEGs between diapause stage at natural low-temperature acclimation (N vs T)of C. italicus egg. F KEGG significant enrichment analysis for DEGs between diapause-terminated stage at natural low-temperature acclimation (N vs T) of C. italicus egg. Table S1. The information of DEGs. Table S2. Functional annotation of Significantly enriched GO at constant low-temperature acclimation (Z vs T). Table S3. Functional annotation of Significantly enriched GO at natural low-temperature acclimation(N vs T). Table S4. KEGG pathway enriched significantly at constant low-temperature acclimation (Z vs T). Table S5. KEGG pathway enriched significantly at natural low-temperature acclimation(N vs T). Table S6. qPCR verification results of transcriptomes. Table S7. Interference verificatio [file 12864_2022_8705_MOESM1_ESM.zip › Supplementary Information/Figure S2,E KEGG significant enrichment analysis for DEGs between diapause stage at natural low-temperature acclimation (N vs T)of C. italicus egg.jpg]

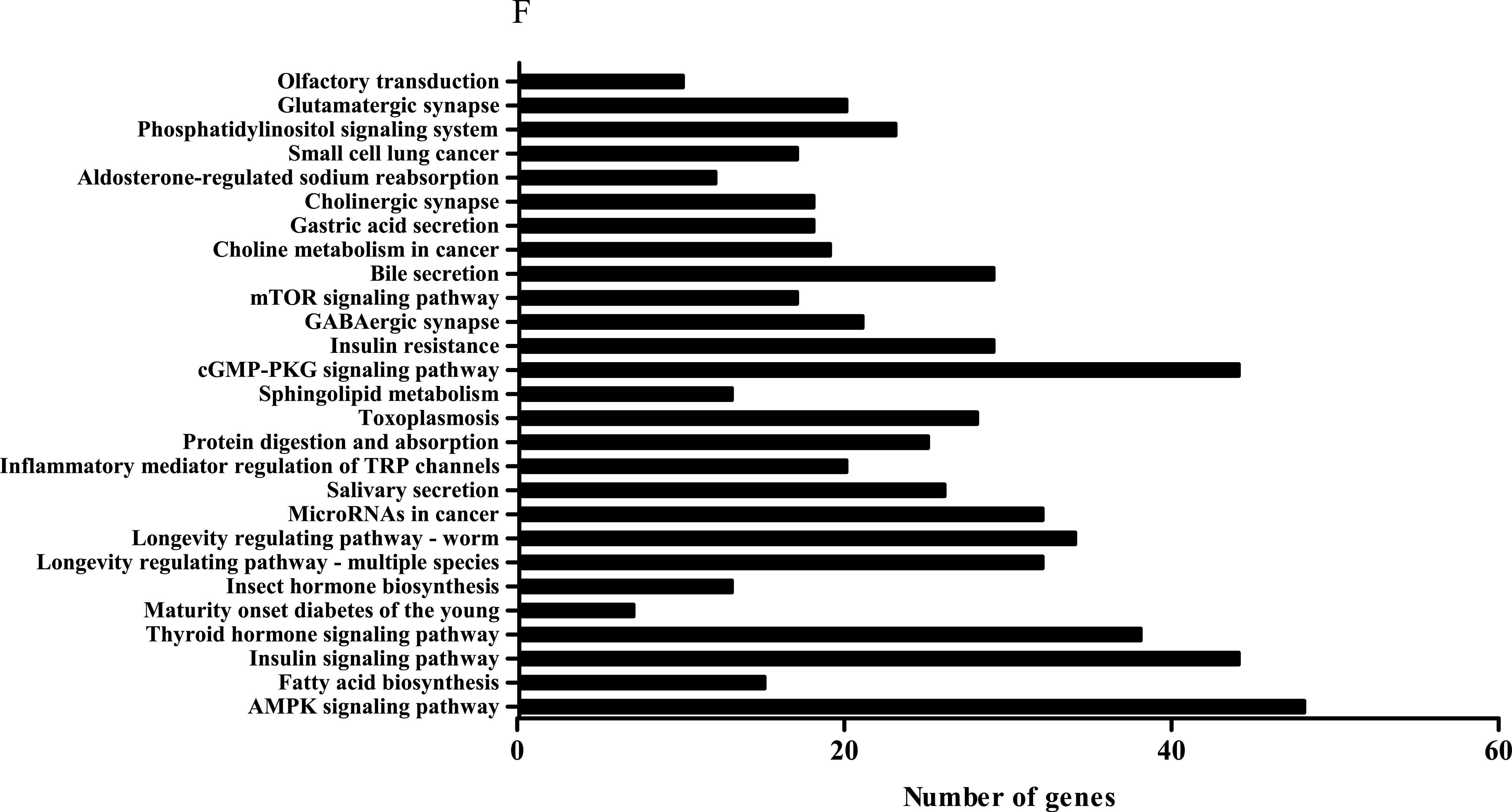

Supplement: Supplementary file 1 — Additional file 1: Figure S1. A Functional annotation of assembled sequences of DEGs of C. italicus egg at constant low-temperature acclimation (Z vs T) based on gene ontology (GO)categorization. Unigenes were annotated in three categories: biological process, cellular components, and molecular functions. B Functional annotation of assembled sequences of DEGs of C. italicus egg at natural low-temperature acclimation (N vs T) based on gene ontology (GO)categorization. Unigenes were annotated in three categories: biological process and molecular functions. Figure S2. A KEGG significant enrichment analysis for DEGs between early-development stage at constant low-temperature acclimation group (Z vs T) of C. italicus egg. B KEGG significant enrichment analysis for DEGs between diapause stage at constant low-temperature acclimation group (Z vs T) of C. italicus egg. C KEGG significant enrichment analysis for DEGs between diapause-terminated stage at constant low-temperature acclimation group (Z vs T) of C. italicus egg. D KEGG significant enrichment analysis for DEGs between early-development stage at natural low-temperature acclimation (N vs T) of C. italicus egg. E KEGG significant enrichment analysis for DEGs between diapause stage at natural low-temperature acclimation (N vs T)of C. italicus egg. F KEGG significant enrichment analysis for DEGs between diapause-terminated stage at natural low-temperature acclimation (N vs T) of C. italicus egg. Table S1. The information of DEGs. Table S2. Functional annotation of Significantly enriched GO at constant low-temperature acclimation (Z vs T). Table S3. Functional annotation of Significantly enriched GO at natural low-temperature acclimation(N vs T). Table S4. KEGG pathway enriched significantly at constant low-temperature acclimation (Z vs T). Table S5. KEGG pathway enriched significantly at natural low-temperature acclimation(N vs T). Table S6. qPCR verification results of transcriptomes. Table S7. Interference verificatio [file 12864_2022_8705_MOESM1_ESM.zip › Supplementary Information/Figure S2,F KEGG significant enrichment analysis for DEGs between diapause-terminated stage at natural low-temperature acclimation (N vs T) of C. italicus egg.jpg]
